# Supplementary material for: Modularity of Zorya defense systems during phage inhibition
Source: Nat Commun. 2025 Mar 8;16:2344. doi: 10.1038/s41467-025-57397-2 (PMC11890865; doi:10.1038/s41467-025-57397-2)
Supplement: Supplementary file 1 — Supplementary Information [file 41467_2025_57397_MOESM1_ESM.pdf]

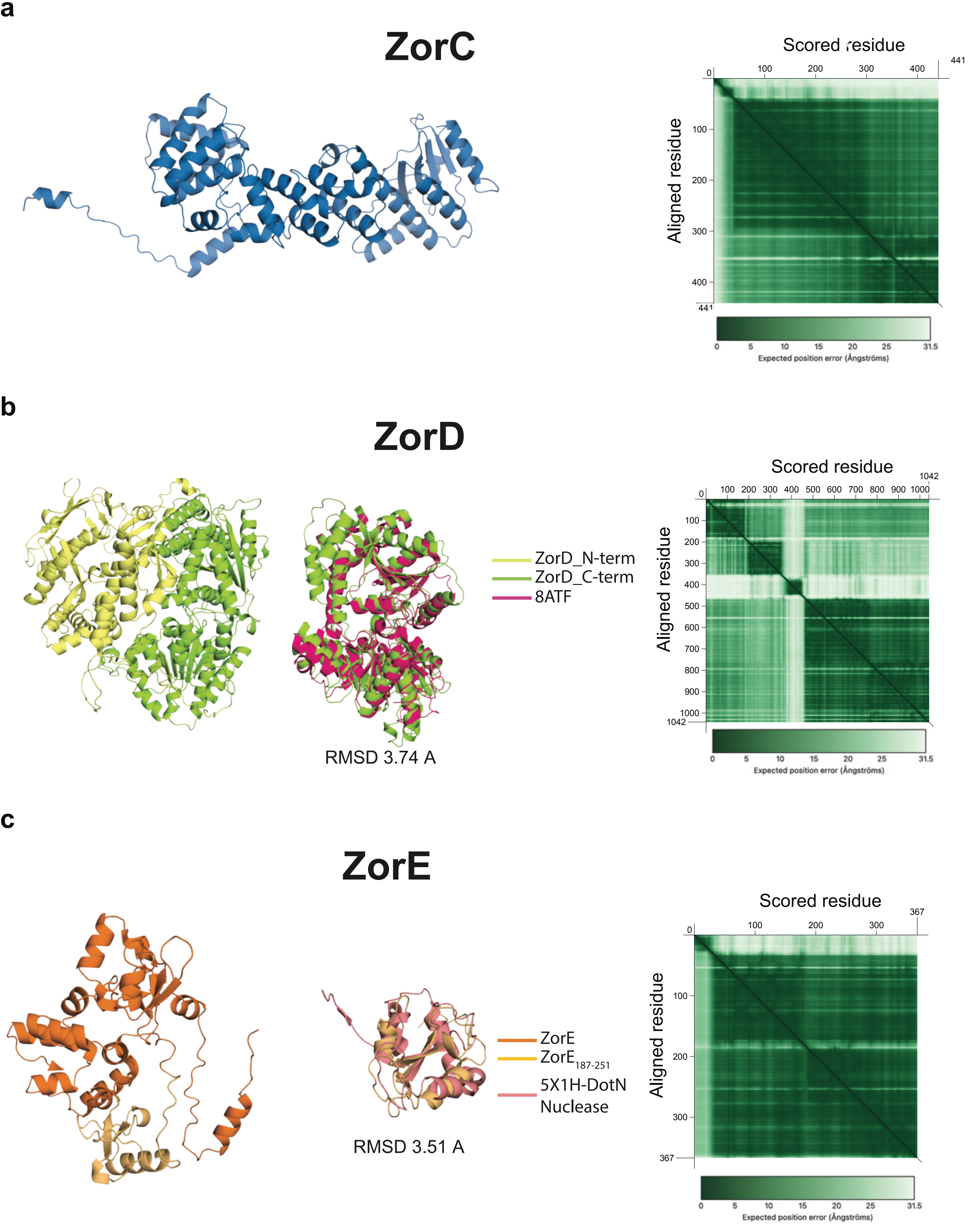
a
ZorC
Scored residue
Aligned residue
Expected position error (Ångströms)
b
ZorD
ZorD_N-term
ZorD_C-term
8ATF
RMSD 3.74 A
Scored residue
Aligned residue
Expected position error (Ångströms)
c
ZorE
ZorE
ZorE187-251
5X1H-DotN Nuclease
RMSD 3.51 A
Scored residue
Aligned residue
Expected position error (Ångströms)

Supplementary Figure 1. Alphafold models of Zorya I and II accessory components. a, Alphafold model and related PAE error for the predicted structure of ZorC. **b**, Alphafold model and related PAE error for the predicted structure of ZorD. ZorD N-terminal and C-terminal domains highlighted in different shades as shown in panel b. The C-terminal domain of ZorD was aligned with its closest homologue, identified through a Foldseek search (PDB: 8ATF, nucleosome-bound Ino80 ATPase).**c,** Alphafold model and related PAE error for the predicted structure of ZorE. $ZorE_{187\text{-}251}$ residues are highlighted in lighter orange. A Foldseek search showed that $ZorE_{187\text{-}251}$ residues align with the DotN nuclease domain (PDB: 5X1H)

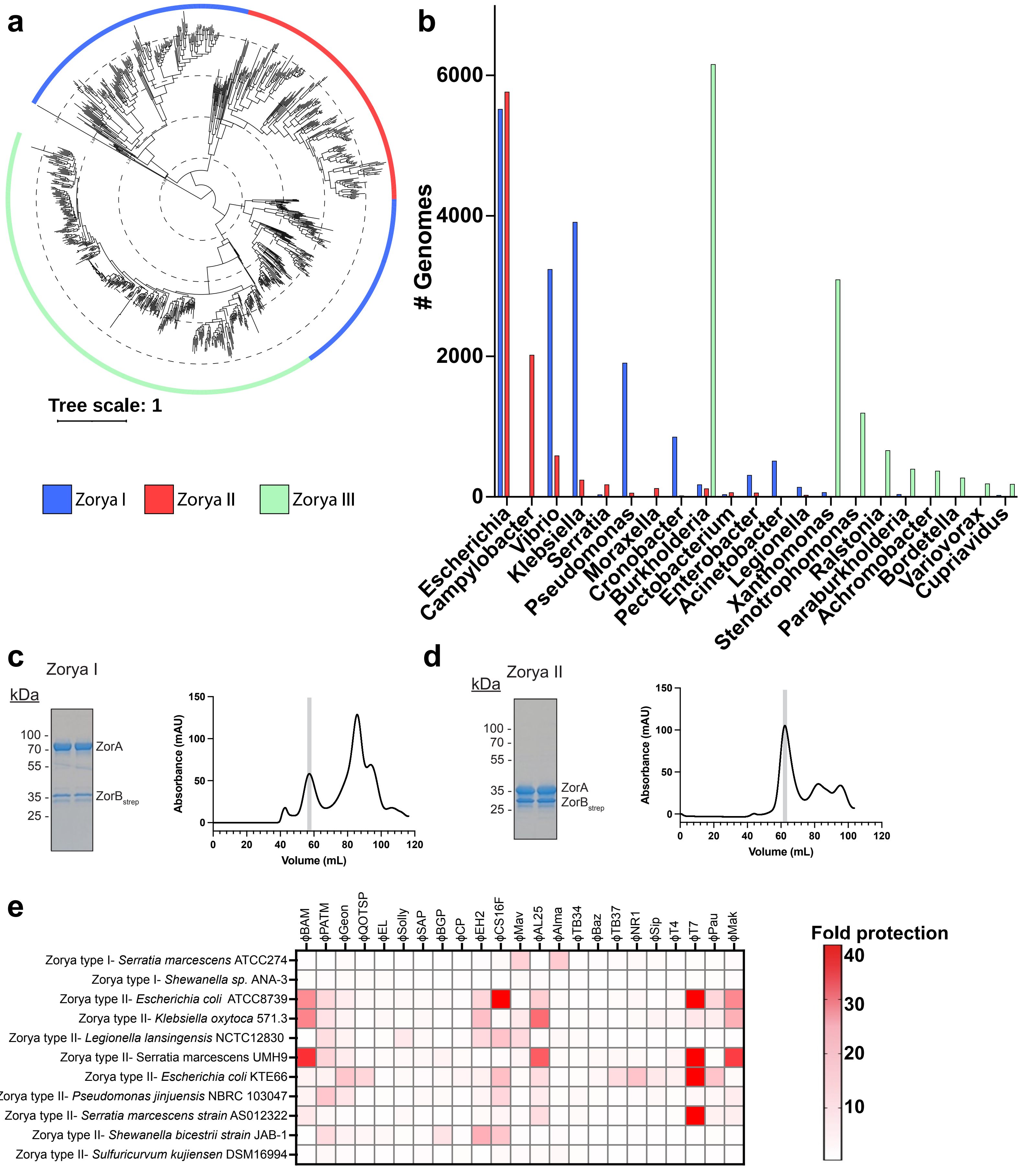
a
Tree scale: 1
Zorya I
Zorya II
Zorya III
b
Genomes
6000
4000
2000
0
Escherichia
Campylobacter
Vibrio
Klebsiella
Serratia
Pseudomonas
Moraxella
Cronobacter
Burkholderia
Pectobacterium
Enterobacter
Acinetobacter
Legionella
Xanthomonas
Stenotrophomonas
Ralstonia
Paraburkholderia
Achromobacter
Bordetella
Variovorax
Cupriavidus
c
Zorya I
kDa
100 -
70 -
55 -
35 -
25 -
ZorA
ZorBstrep
Absorbance (mAU)
150
100
50
0
0 20 40 60 80 100 120
Volume (mL)
d
Zorya II
kDa
100 -
70 -
55 -
35 -
25 -
ZorA
ZorBstrep
Absorbance (mAU)
150
100
50
0
0 20 40 60 80 100 120
Volume (mL)
e
ϕBAM ϕPATM ϕGeon ϕQOTSP ϕEL ϕSolly ϕSAP ϕBGP ϕCP ϕEH2 ϕCS16F ϕMav ϕAL25 ϕAlma ϕTB34 ϕBaz ϕTB37 ϕNR1 ϕSip ϕT4 ϕT7 ϕPau ϕMak
Zorya type I- Serratia marcescens ATCC274
Zorya type I- Shewanella sp. ANA-3
Zorya type II- Escherichia coli ATCC8739
Zorya type II- Klebsiella oxytoca 571.3
Zorya type II- Legionella lansingensis NCTC12830
Zorya type II- Serratia marcescens UMH9
Zorya type II- Escherichia coli KTE66
Zorya type II- Pseudomonas jinjuensis NBRC 103047
Zorya type II- Serratia marcescens strain AS012322
Zorya type II- Shewanella bicestrii strain JAB-1
Zorya type II- Sulfuricurvum kujiensen DSM16994
Fold protection
40
30
20
10

Supplementary Figure 2. a, Phylogenetic tree based on the concatenated alignments of the sequences of ZorA and ZorB from each Zorya subtype. **b,** Bar-chart summarising the phylogenetic distribution of Zorya I, Zorya II and Zorya III. **c,** SDS-PAGE gel and size-exclusion chromatogram of purified Shewanella sp. strain ANA-3 ZorAB. **d,** SDS-PAGE gel and size-exclusion chromatogram of purified Sulfuricurvum kujiense ZorAB. Grey boxes represent fractions used for structural studies. **e,**The anti-phage activity of Zorya I and Zorya II homologues was evaluated by calculation of their fold protection against a suite of coliphages when over-expressed in *E. coli* MT56. Fold protection was calculated by dividing the value of efficiency of plating (EOP) for strains expressing each tested homologue by the EOP value of a strain carrying the empty vector (pGM39), when infected with phages as shown in the figure**.**

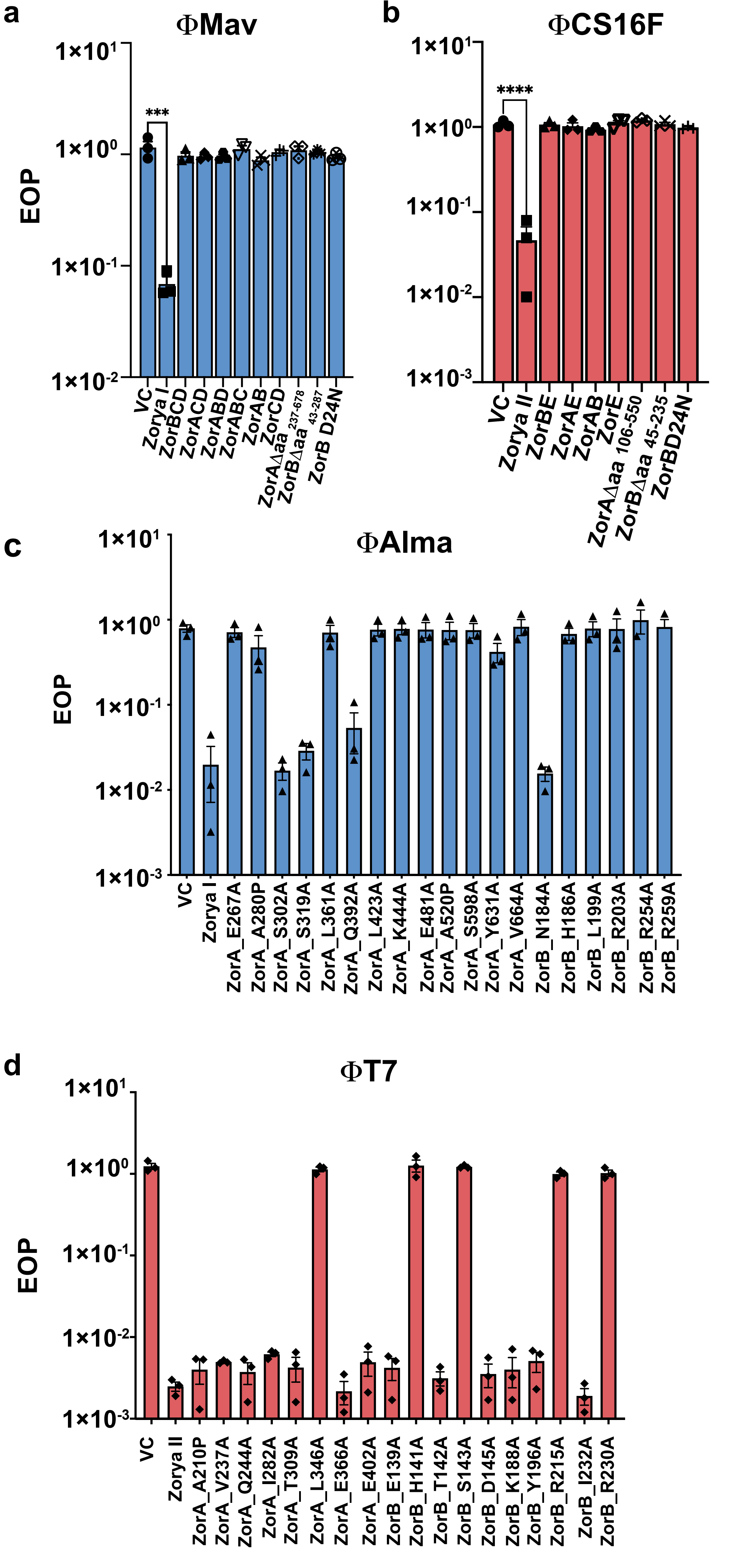

a
ΦMav
EOP
***
VC
Zorya I
ZorBCD
ZorACD
ZorABD
ZorABC
ZorAB
ZorCD
ZorAΔaa 237-678
ZorBΔaa 43-287
ZorB D24N
b
ΦCS16F
****
VC
Zorya II
ZorBE
ZorAE
ZorAB
ZorE
ZorAΔaa 106-550
ZorBΔaa 45-235
ZorBD24N
c
ΦAlma
EOP
VC
Zorya I
ZorA_E267A
ZorA_A280P
ZorA_S302A
ZorA_S319A
ZorA_L361A
ZorA_Q392A
ZorA_L423A
ZorA_K444A
ZorA_E481A
ZorA_A520P
ZorA_S598A
ZorA_Y631A
ZorA_V664A
ZorB_N184A
ZorB_H186A
ZorB_L199A
ZorB_R203A
ZorB_R254A
ZorB_R259A
d
ΦT7
EOP
VC
Zorya II
ZorA_A210P
ZorA_V237A
ZorA_Q244A
ZorA_I282A
ZorA_T309A
ZorA_L346A
ZorA_E366A
ZorA_E402A
ZorB_E139A
ZorB_H141A
ZorB_T142A
ZorB_S143A
ZorB_D145A
ZorB_K188A
ZorB_Y196A
ZorB_R215A
ZorB_I232A
ZorB_R230A

Supplementary Figure 3. a, Efficiency of plating (EOP) measurement for *E. coli* MT56 carrying empty vector (VC, pGM39) or the same plasmid encoding Zorya I, ZorA I, ZorB I, ZorABC, ZorABD, ZorBDC, ZorAB I, ZorCD, ZorB D24N, ZorA IΔaa237-696 and ZorB IΔaa43-287 when infected with phage ϕMav. Points show mean +/- SEM (n = 3 biological replicates). **b,** Efficiency of plating (EOP) measurement for *E. coli* MT56 carrying empty vector (VC, pGM39) or the same plasmid encoding Zorya II, ZorA II, ZorB II, ZorAB II, ZorE, ZorB D24N, ZorA IΔaa106-550 and ZorB IΔaa45-235 when infected with phage ϕCS16F. Points show mean +/- SEM (n = 3 biological replicates). **c,** Efficiency of plating (EOP) measurement for *E. coli* MT56 harbouring empty vector (VC, pGM39), the same plasmid encoding wild-type Zorya I or a version of Zorya I where single point mutations were introduced in ZorA I or ZorB I, as indicated in panel **c,** when infected with ϕAlma**. d,** Efficiency of plating (EOP) measurement for *E. coli* MT56 expressing empty vector (VC, pGM39), the same plasmid encoding wild-type Zorya II or a version of Zorya II carrying point mutations in ZorA II or ZorB II, as indicated in panel **d,** when infected with ϕT7**.** Induction of each construct was performed by addition of 0.02% *L*-Rhamnose. For all panels, points show mean +/- SEM (n = 3 biological replicates). Statistical significance for each panel was calculated with Graphpad applying a one-way ANOVA with Dunnett's multiple comparison test. No significance was detected, unless indicated (*p≤ 0.05). For Panel **h** and **i,** the statistical analysis results is reported in Table S4.

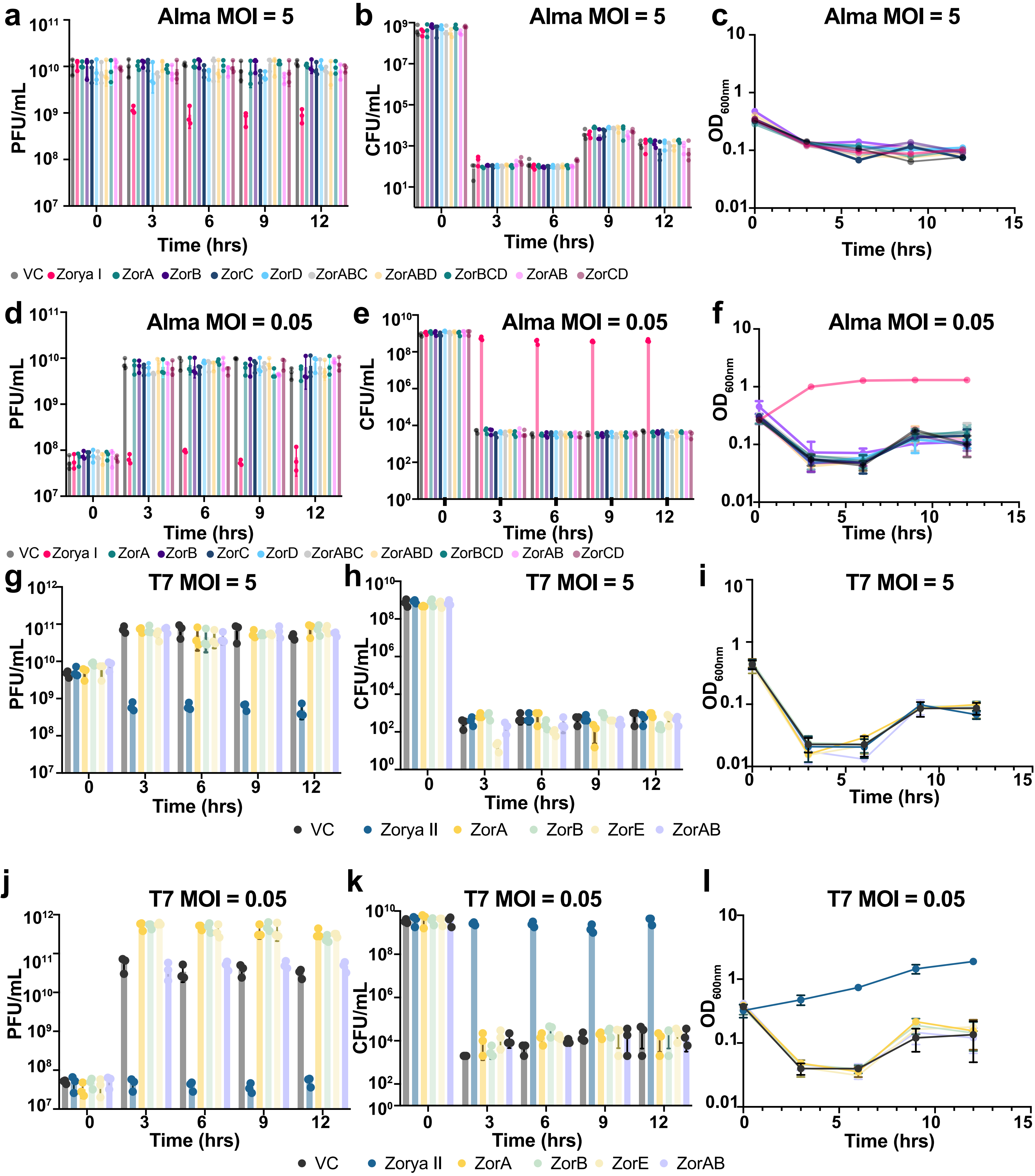

a
Alma MOI = 5
PFU/mL
Time (hrs)
b
Alma MOI = 5
CFU/mL
Time (hrs)
c
Alma MOI = 5
OD600nm
Time (hrs)
VC Zorya I ZorA ZorB ZorC ZorD ZorABC ZorABD ZorBCD ZorAB ZorCD
d
Alma MOI = 0.05
PFU/mL
Time (hrs)
e
Alma MOI = 0.05
CFU/mL
Time (hrs)
f
Alma MOI = 0.05
OD600nm
Time (hrs)
VC Zorya I ZorA ZorB ZorC ZorD ZorABC ZorABD ZorBCD ZorAB ZorCD
g
T7 MOI = 5
PFU/mL
Time (hrs)
h
T7 MOI = 5
CFU/mL
Time (hrs)
i
T7 MOI = 5
OD600nm
Time (hrs)
VC Zorya II ZorA ZorB ZorE ZorAB
j
T7 MOI = 0.05
PFU/mL
Time (hrs)
k
T7 MOI = 0.05
CFU/mL
Time (hrs)
l
T7 MOI = 0.05
OD600nm
Time (hrs)
VC Zorya II ZorA ZorB ZorE ZorAB

Supplementary Figure 4. Zorya I and Zorya II act through population wide immunity. *E. coli* MT56 harbouring plasmids as shown in panel **a-f** were grown in LB supplemented with 0.02% *L*-Rhamnose and infected with ϕAlma at MOI 5 or 0.05. The **a,d,** titre (PFU/mL), **b,e,** cell counts (CFU/mL) and **c,f** the growth rate (OD_{600nm}) of each culture was measured at several timepoints, as shown in panels **a-f**, over the course of 12 hrs post infection. **g-l,** *E. coli* MT56 carrying VC or Zorya II were grown in LB supplemented with 0.02% *L*-Rhamnose and infected with ϕT7 at MOI 5 or 0.05. The **g,j,** titre (PFU/mL), **h,k,** cell counts (CFU/mL) and **i,l,** the growth rate (OD_{600nm}) of each culture was measured at several timepoints, as shown in panels **g-l**, over the course of 12 hrs post infection. For all panels, points show mean +/- SEM (n = 3 biological replicates). Statistical significance was calculated with Graphpad applying a one-way ANOVA with Dunnett's multiple comparison test. No significance was detected, unless indicated (*p≤ 0.05).

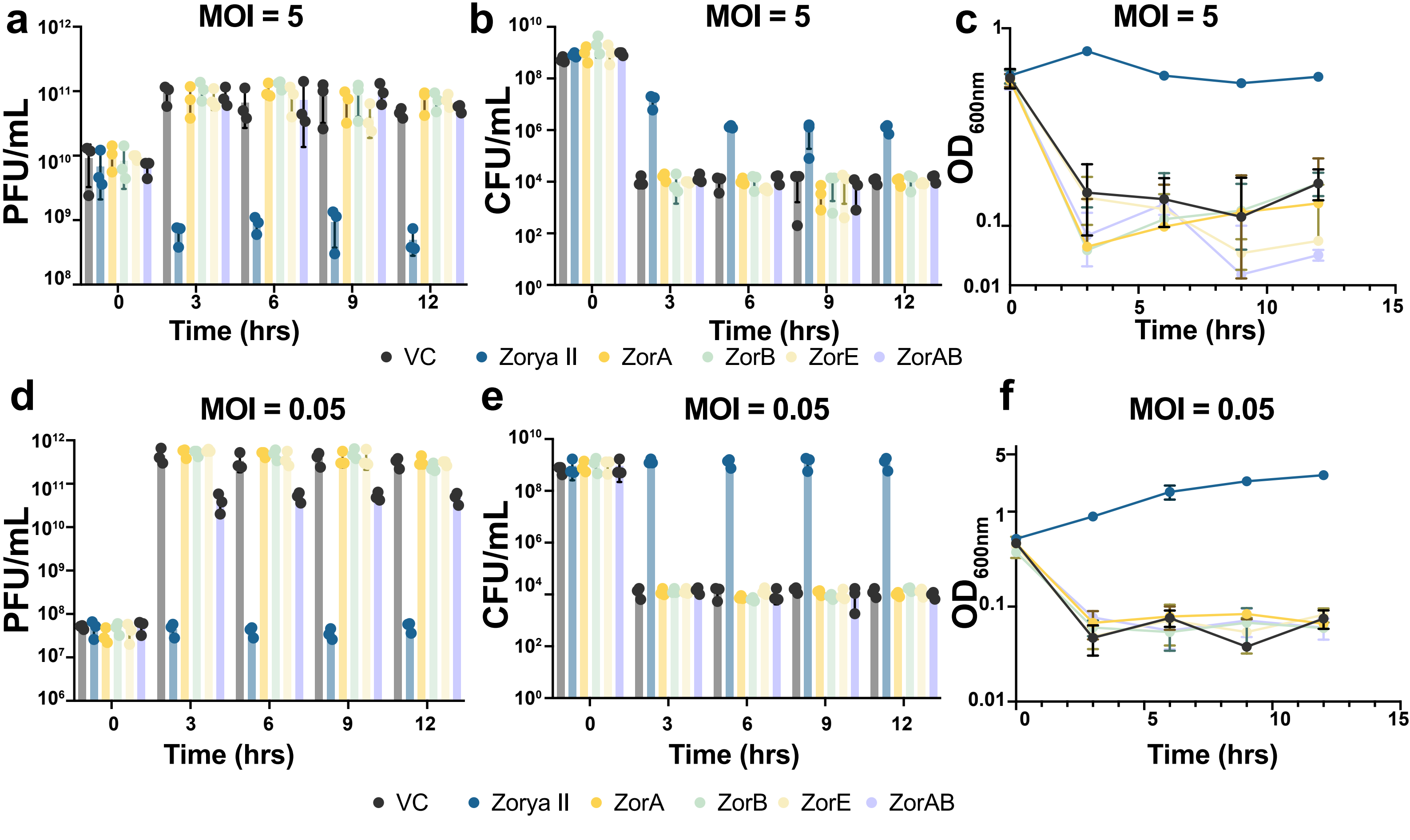

a
MOI = 5
PFU/mL
Time (hrs)
b
MOI = 5
CFU/mL
Time (hrs)
c
MOI = 5
OD600nm
Time (hrs)
VC
Zorya II
ZorA
ZorB
ZorE
ZorAB
d
MOI = 0.05
PFU/mL
Time (hrs)
e
MOI = 0.05
CFU/mL
Time (hrs)
f
MOI = 0.05
OD600nm
Time (hrs)
VC
Zorya II
ZorA
ZorB
ZorE
ZorAB

Supplementary Figure 5. Native expression of Zorya II mediates population-wide immunity in response to phage infection. *E. coli* MT56 harbouring empty vector (VC, pSUPROM) or the same vector carrying Zorya II or its mutants under the control of their native promoter as shown in panel **a-f** were infected with ϕphAvM at MOI 5 or 0.05. The **a,d,** titre (PFU/mL), **b,e,** cell counts (CFU/mL) and **c,f** the growth rate (OD_{600nm}) of each culture was measured at several timepoints, as shown in panels **a-f**, over the course of 12 hrs post infection. For all panels, points show mean +/- SEM (n = 3 biological replicates).

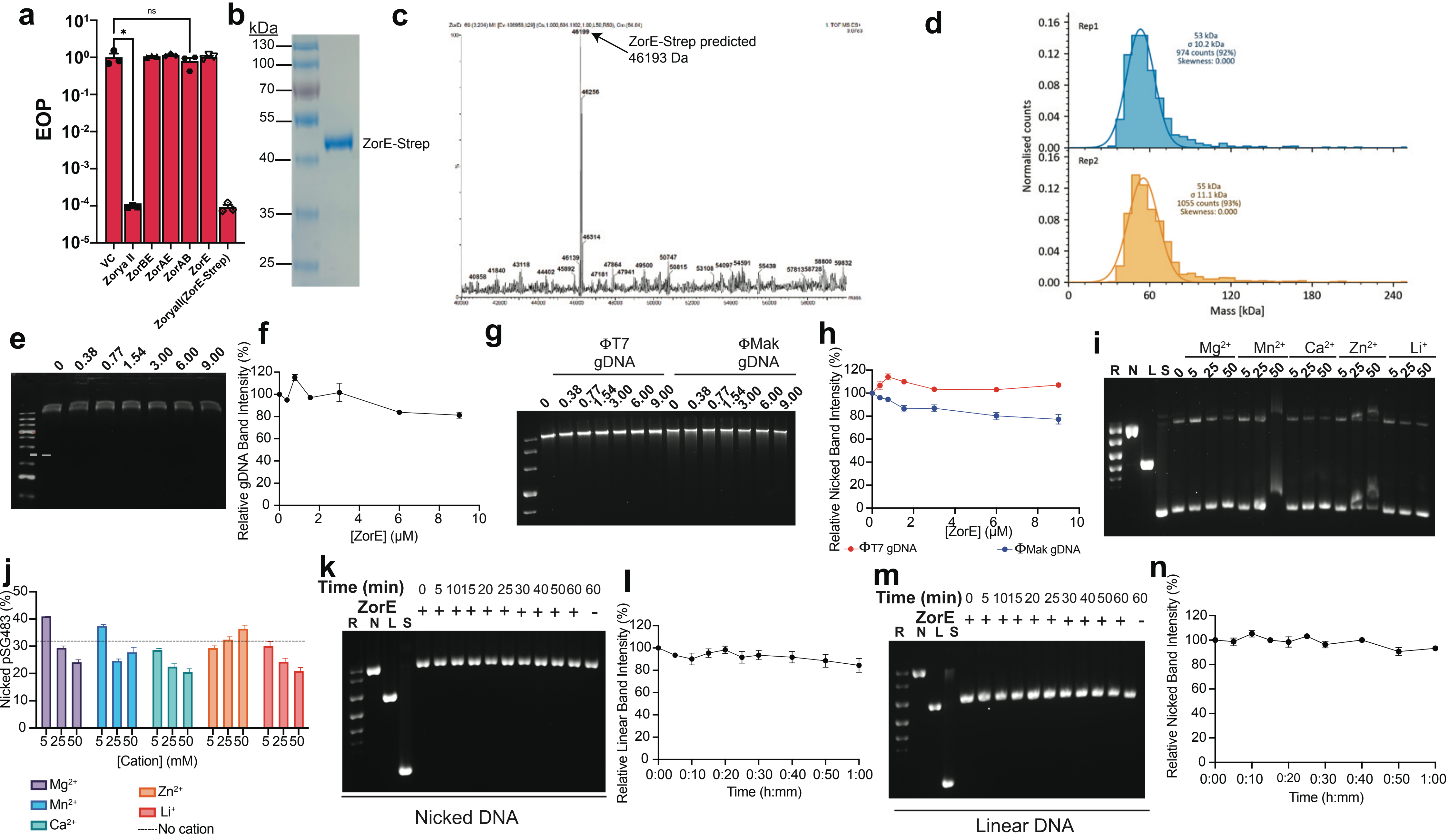

a
EOP
VC
Zorya II
ZorBE
ZorAE
ZorAB
ZorE
ZoryaII(ZorE-Strep)
ns
*
b
kDa
130
100
70
55
40
35
25
ZorE-Strep
c
ZorE-Strep predicted
46193 Da
d
Rep1
53 kDa
σ 10.2 kDa
974 counts (92%)
Skewness: 0.000
Rep2
55 kDa
σ 11.1 kDa
1055 counts (93%)
Skewness: 0.000
Normalised counts
Mass [kDa]
e
0 0.38 0.77 1.54 3.00 6.00 9.00
f
Relative gDNA Band Intensity (%)
[ZorE] (μM)
g
ΦT7 gDNA
ΦMak gDNA
h
Relative Nicked Band Intensity (%)
[ZorE] (μM)
ΦT7 gDNA
ΦMak gDNA
i
R N L S 0
Mg2+ Mn2+ Ca2+ Zn2+ Li+
5 25 50
j
Nicked pSG483 (%)
[Cation] (mM)
Mg2+
Mn2+
Ca2+
Zn2+
Li+
No cation
k
Time (min) 0 5 10 15 20 25 30 40 50 60 60
ZorE
R N L S
Nicked DNA
l
Relative Linear Band Intensity (%)
Time (h:mm)
m
Time (min) 0 5 10 15 20 25 30 40 50 60 60
ZorE
R N L S
Linear DNA
n
Relative Nicked Band Intensity (%)
Time (h:mm)

Supplementary Figure 6. ZorE is a monomeric nickase with variable effects of metals. **a,** Efficiency of plating (EOP) measurement for *E. coli* MT56 carrying empty vector (VC, pGM39) or the same plasmid encoding Zorya II, ZorA II, ZorB II, ZorAB II, ZorE or ZorAII+ZorBII+ZorE-Strep when infected with ϕT7. **b,** SDS-PAGE of the final purified ZorE-Strep showing a single band at the expected size. **c,** Size exclusion chromatogram showing the expected elution volume of monomeric ZorE-Strep based on molecular weight (dashed line) and the peak collected for the final purified ZorE-Strep (blue). **d,** Mass photometry (Refeyn) for the ZorE-Strep sample shows a single population in solution representing monomeric ZorE-Strep. **e,** ZorE was titrated against constant *E.* coli MG1655 gDNA (200 ng). Reactions were incubated for 60 min at 37 °C in the presence of 5 mM $Mg^{2+.}$ **f,** Densitometry analysis of nicking of *E.* coli MG1655 gDNA by ZorE as shown in **panel e**. **g,** ZorE was titrated against constant ϕT7and ϕMak gDNA (200 ng). Reactions were incubated for 60 min at 37 °C in the presence of 5 mM Mg^{2+}. **h,** Densitometry analysis of nicking of ϕT7 and ϕMak gDNA by ZorE as shown in **panel g**. **i,** ZorE (768 nM) was incubated with supercoiled pSG483 plasmid DNA (6 nM) in presence of various metal cations. Cations were tested at 5, 25, and 50 mM. Reactions were incubated for 60 min at 37 °C. **j,** Densitometry analysis of nicking of pSG483 by ZorE in presence of various cations as shown in **panel i.** **k,** ZorE (768 nM) was incubated with linearised plasmid pSG483 (6 nM) at 37 °C for 0 to 60 min with 5 mM Mg^{2+}. **l,** Densitometry analysis of nicking of linear plasmid pSG483 by ZorE as shown in **panel k**. **m,** ZorE (768 nM) was incubated with nicked plasmid pSG483 (6 nM) at 37 °C for 0 to 60 min with 5 mM Mg^{2+}. **n,** Densitometry analysis of nicking of nicked plasmid pSG483 by ZorE as shown in **panel m**. For panels **e,g,i,k,m,** reactions were stopped by the addition of EDTA and SDS and products were analysed by gel electrophoresis in a 1x TAE, 1.4% agarose gel, post-stained with ethidium bromide. In all gels, control lanes represent forms of plasmid pSG483; R, relaxed (multiple topoisomers); N, nicked; L, linear; S, supercoiled. For panels **f,h,j,l,n** densitometry was performed using ImageJ (version 1.54g) with background subtracted and band intensity measured in triplicate. The percentage nicked, linear, and supercoiled pSG483 DNA of the total pSG483 DNA per lane was determined by calculating the average intensity (n = 3) of each lanes' nicked, linear, and supercoiled bands, respectively. as a percentage of the total average intensity of all bands per lane. Relative band intensity was determined by normalising the average (n = 3) intensity of the "0 µM ZorE" lane to 100% and taking the average intensity of the subsequent lanes' bands as a percentage of the "0 µM ZorE" lane. Error bars represent the standard error of the mean of triplicate data.

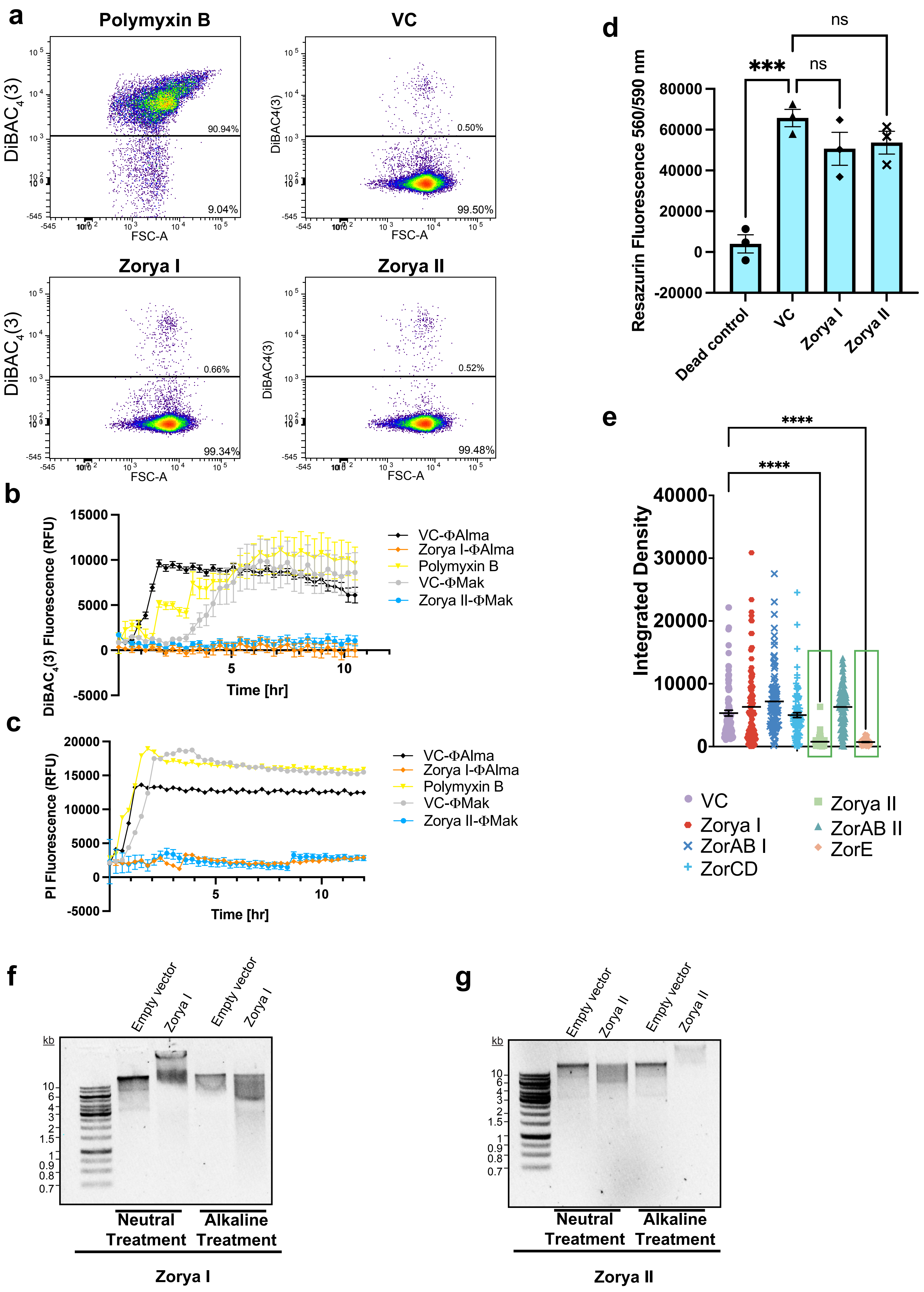

a
Polymyxin B
VC
Zorya I
Zorya II
DiBAC4(3)
FSC-A
90.94%
9.04%
0.50%
99.50%
0.66%
99.34%
0.52%
99.48%
b
DiBAC4(3) Fluorescence (RFU)
Time [hr]
VC-ΦAlma
Zorya I-ΦAlma
Polymyxin B
VC-ΦMak
Zorya II-ΦMak
c
PI Fluorescence (RFU)
Time [hr]
d
Resazurin Fluorescence 560/590 nm
***
ns
ns
Dead control
VC
Zorya I
Zorya II
e
Integrated Density
****
****
VC
Zorya I
ZorAB I
ZorCD
Zorya II
ZorAB II
ZorE
f
Empty vector
Zorya I
kb
Neutral Treatment
Alkaline Treatment
Zorya I
g
Empty vector
Zorya II
kb
Neutral Treatment
Alkaline Treatment
Zorya II

Supplementary Figure 7. Zorya I and Zorya II do not alter the membrane potential and metabolism of cells. a, Analysis of *E. coli* MT56 carrying empty vector (VC, pGM39) or the same plasmid encoding Zorya I or Zorya II by flow cytometry following induction with 0.2% *L*-Rhamnose. As a positive control, cells carrying VC were treated with 5 µg/mL of polymyxin B. Staining with $DiBAC_4(3)$ was used to assess loss of membrane potential. The image is representative of 3 independent experiments. **b-c,** Kinetic reads of **(b)** $DiBAC_4(3)$ fluorescence or **(c)** propidium iodide fluorescence for *E. coli* MT56 carrying empty vector (VC, pGM39) or a plasmid encoding Zorya I when infected with ϕAlma, or for cells harbouring VC or a plasmid encoding Zorya II when infected with ϕMak. Fluorescence was recorded every 20 min over the course of 12 hr. **d,** Strains as in **panel a-c,** were grown for 2 hrs at 37 °C in presence of 0.2% *L*-Rhamnose and subsequently treated with CellTiter Blue stain (Promega) to assess changes in their metabolism. Cells carrying VC were incubated 10 min at 100 °C as a positive control. Changes in the metabolism of each sample was assessed by measuring the ratio fluorescence at 560nm and at 590nm. For panels **b-d,** points show mean +/- SEM (n = 3 biological replicates).**e,** Quantification of the integrated density (sum of pixel values over single-cells area) for DAPI staining in Figure **5g.** The integrated density was quantified using Fiji. Point show mean +/- SEM (n = 100 cells). Statistical analysis was performed using non-parametric Kruskal-Wallis Test in GraphPad 9. Significance was reported as $p \leq 0.05$. **f-g** *E. coli* MT56 harbouring empty vector (VC, pGM39) or the same plasmid encoding Zorya I or Zorya II were grown until exponential phase and then infected with MOI 0.1 of ϕAlma (**f**) or ϕT7 **(g).** Cells were retrieved after first burst event and total genomic DNA extracted. Genomic DNA was subjected to neutral and alkaline treatment, as described in Material and Methods, and subjected to electrophoretic analysis. For panels **f-g**, images are representative of three independent experiments.

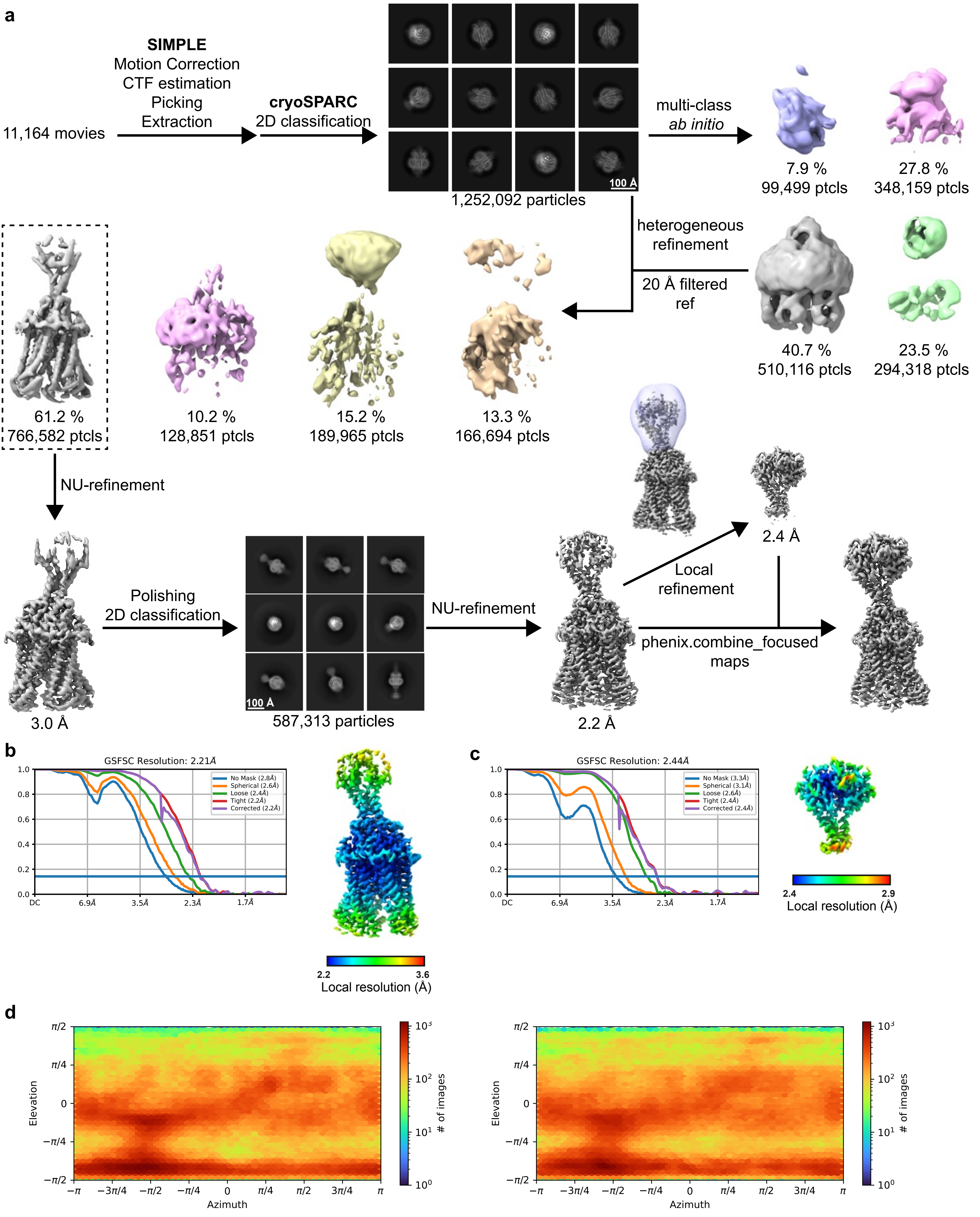

a
SIMPLE
Motion Correction
CTF estimation
Picking
Extraction
11,164 movies
cryoSPARC
2D classification
100 Å
1,252,092 particles
multi-class
ab initio
7.9 %
99,499 ptcls
27.8 %
348,159 ptcls
heterogeneous
refinement
20 Å filtered
ref
40.7 %
510,116 ptcls
23.5 %
294,318 ptcls
61.2 %
766,582 ptcls
10.2 %
128,851 ptcls
15.2 %
189,965 ptcls
13.3 %
166,694 ptcls
NU-refinement
3.0 Å
Polishing
2D classification
100 Å
587,313 particles
NU-refinement
2.2 Å
Local
refinement
2.4 Å
phenix.combine_focused
maps
b
GSFSC Resolution: 2.21Å
No Mask (2.8Å)
Spherical (2.6Å)
Loose (2.4Å)
Tight (2.2Å)
Corrected (2.2Å)
DC
6.9Å
3.5Å
2.3Å
1.7Å
2.2
3.6
Local resolution (Å)
c
GSFSC Resolution: 2.44Å
No Mask (3.3Å)
Spherical (3.1Å)
Loose (2.6Å)
Tight (2.4Å)
Corrected (2.4Å)
2.4
2.9
Local resolution (Å)
d
Elevation
Azimuth
# of images
π/2
π/4
0
−π/4
−π/2
−π
−3π/4
−π/2
−π/4
0
π/4
π/2
3π/4
π

Supplementary Figure 8. Cryo-EM processing workflow of *Shewanella* sp. strain ANA-3 type I ZorA$_5$B$_2$ complex with local and global resolution estimates. **a**, Image processing workflow. **b**, Left, gold-standard FSC curves used for global-resolution estimate of the consensus volume as calculated within cryoSPARC. Right, local resolution estimate of the consensus volume as determined within Relion. **c**, Left, Gold-standard FSC curves used for global-resolution estimate of the ZorB peptidoglycan-binding domain dimer volume, derived from local refinement, as calculated within cryoSPARC. Right, local resolution estimate of the peptidoglycan-binding domain volume, derived from local refinement, as determined within Relion. **d,** angular distribution plot of the consensus (left) or peptidoglycan-binding domain (right) particle sets.

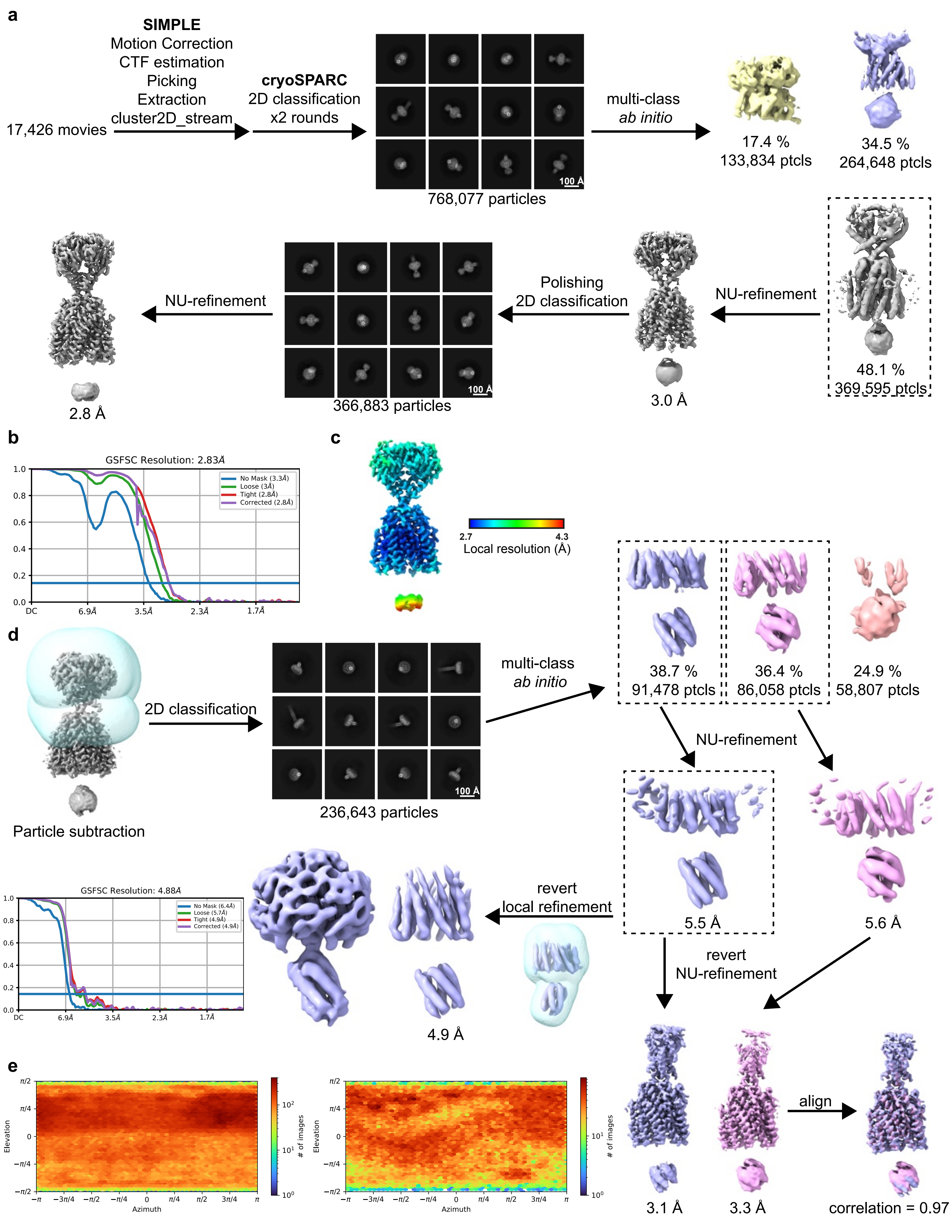
a
SIMPLE
Motion Correction
CTF estimation
Picking
Extraction
cluster2D_stream
17,426 movies
cryoSPARC
2D classification
x2 rounds
100 Å
768,077 particles
multi-class
ab initio
17.4 %
133,834 ptcls
34.5 %
264,648 ptcls
48.1 %
369,595 ptcls
NU-refinement
3.0 Å
Polishing
2D classification
100 Å
366,883 particles
NU-refinement
2.8 Å
b
GSFSC Resolution: 2.83Å
No Mask (3.3Å)
Loose (3Å)
Tight (2.8Å)
Corrected (2.8Å)
c
2.7
4.3
Local resolution (Å)
d
Particle subtraction
2D classification
100 Å
236,643 particles
multi-class
ab initio
38.7 %
91,478 ptcls
36.4 %
86,058 ptcls
24.9 %
58,807 ptcls
NU-refinement
5.5 Å
5.6 Å
revert
local refinement
4.9 Å
GSFSC Resolution: 4.88Å
No Mask (6.4Å)
Loose (5.7Å)
Tight (4.9Å)
Corrected (4.9Å)
revert
NU-refinement
3.1 Å
3.3 Å
align
correlation = 0.97
e
Elevation
Azimuth
# of images

Supplementary Figure 9. Cryo-EM processing workflow of *Sulfuricurvum kujiense* type II ZorA$_5$B$_2$ complex with local and global resolution estimates.a, Image processing workflow. **b**, Gold-standard FSC curves used for global-resolution estimate as calculated within cryoSPARC. **c**, Local resolution estimate as determined within Relion. **d**, Additional processing steps used to improved density for the cytoplasmic extensions of *S. kujiense* ZorAB. **e,** angular distribution plot of the full complex (left) or cytoplasmic extension focused (right) particle sets.

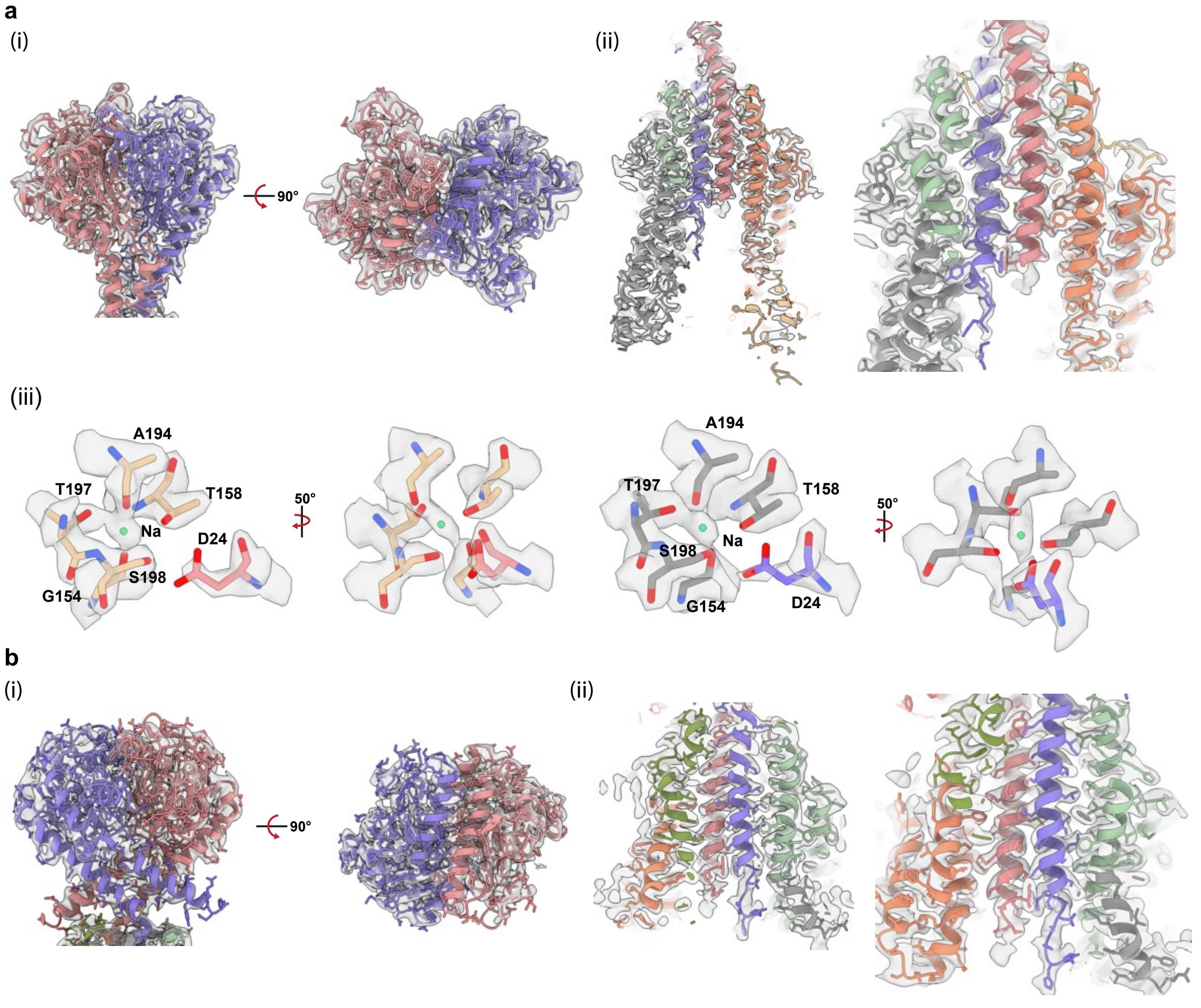

a
(i)
90°
(ii)
(iii)
A194
T197
T158
Na
D24
S198
G154
50°
A194
T197
T158
S198
Na
G154
D24
50°
b
(i)
90°
(ii)

Supplementary Figure 10. Model-to-map fits of key structural elements.a, Model overlayed with density for *Shewanella* sp. strain ANA-3 type I $ZorA_5B_2$ complex, depicting (i) the ZorB peptidoglycan-binding domain (composite map; contour level of 10.8), (ii) a slice through of the core complex shown at lower (left) or higher (right) magnification (composite map; contour level of 10.8) or (iii) the sodium-binding sites (left, site 1; right, site 2) at two viewing angles (sharpened map; contour level of 1.22). **b,** Model overlayed with density for *Sulfuricurvum kujiense* type II $ZorA_5B_2$ complex, depicting (i) the ZorB peptidoglycan-binding domain or (ii) a slice through of the core complex shown at lower (left) or higher (right) magnification (all depictions used a map contour level of 0.25)

Supplementary Table 1. Strains and plasmids used in this study

Name	Description	Reference
**<u>Strains</u>**		
Escherichia coli		
Mt56(DE3)	BL21(DE3) derivative optimised for expression of membrane proteins	[1]
DH5α	Cloning strain, F– φ80lacZΔM15 Δ(lacZYA-argF)U169 recA1 endA1 hsdR17(rK–, mK+) phoA supE44 λ–thi-1 gyrA96 relA1	New England Biolabs
Serratia marcescens		
Serratia marcescens		
S. marcescens ATCC 274	*S. marcescens* strain ATCC 274	NCTC
**Plasmids**		
pT12-ecMotAB	Rhamnose-inducible expression vector (Kn^R); carrying the coding sequence for MotAB from *E.coli* W. A TEV cleavage site and 2xStrepII tag are encoded directly downstream and in-frame with MotB.	[2]
pGM39	Rhamnose-inducible expression vector (Kn^R) where the ecMotAB-TEV-2xStrepII tag insert has been deleted by KLD, to leave an empty multiple cloning site.	
pGM15	Coding sequence of ZorAB II from *Sulfuricurvum kujiense* DSM 16994 (SULKU_RS11880 and SULKU_RS11885) in	This study

	pT12-derived plasmid. Insert was cloned in pT12-ecMotAB to replace ecMotAB and in frame with the TEV site and 2xStrepII tag	
pGM99	Coding sequence of Zorya I operon from *Shewanella sp.* ANA-3 (SHEWANA3_RS19785 SHEWANA3_RS19790 and SHEWANA3_RS19795) in pGM39	This study
pGM54	Coding sequence of ZorAB I from *Shewanella sp.* ANA-3 in pT12-derived plasmid. Insert was cloned in pT12-ecMotAB to replace ecMotAB and in frame with the TEV site and 2xStrepII tag	This study
pGM23	Coding sequence of Zorya II operon from *E. coli* ATCC 8739 (ECOLC_RS20900, ECOLC_RS20905 and ECOLC_RS20910) in pGM39	This study
pGM35	Coding sequence of ZorBE operon from *E. coli* ATCC 8739 (ECOLC_RS20900, ECOLC_RS20905 and ECOLC_RS20910) in pGM39	This study
pGM36	Coding sequence of ZorAE operon from *E. coli* ATCC 8739 (ECOLC_RS20900, ECOLC_RS20905 and ECOLC_RS20910) in pGM39	This study
pGM37	Coding sequence of ZorE from *E. coli* ATCC 8739 in pGM39	This study
pGM38	Coding sequence of ZorAB II from *E. coli* ATCC 8739 in pGM39	This study

pGM46	Coding sequence of Zorya II from *E. coli* ATCC 8739 in pGM39, with ZorB II carrying a D24N mutation.	This study
pGM55	Coding sequence of Zorya II from *E. coli* ATCC 8739 in pGM39, with ZorA II carrying a deletion from aa106 to aa550(cytoplasmic domain)	This study
pGM56	Coding sequence of Zorya II from *E. coli* ATCC 8739 in pGM39, with ZorB II carrying a deletion from aa45 to aa235 (periplasmic domain)	This study
pGM70	Coding sequence of Zorya I from *S. marcescens* ATCC 274(SMATCC274_RS21230, SMATCC274_RS21235 SMATCC274_RS21240 and SMATCC274_RS21245) in pGM39	This study
pGM85	Coding sequence of ZorCD from *S. marcescens* ATCC 274 in pGM39	This study
pGM86	Coding sequence of ZorAB I from *S. marcescens* ATCC 274 in pGM39	This study
pGM124	Coding sequence of ZorABC from *S. marcescens* ATCC 274 in pGM39	This study
pGM125	Coding sequence of ZorBCD from *S. marcescens* ATCC 274 in pGM39	This study
pGM140	Coding sequence of Zorya I *S. marcescens* ATCC 274 in pGM39, with ZorB I carrying a deletion a D24N mutation	This study
pGM141	Coding sequence of ZorABD from *S. marcescens* ATCC 274 in pGM39	This study
pGM142	Coding sequence of ZorACD from *S. marcescens* ATCC 274 in pGM39	This study

pGM223	Coding sequence of Zorya I *S. marcescens* ATCC 274 in pGM39, with ZorA I carrying a deletion from aa237 to aa678 (cytoplasmic domain)	This study
pGM224	Coding sequence of Zorya I *S. marcescens* ATCC 274 in pGM39, with ZorB I carrying a deletion from aa43 to aa287 (periplasmic domain)	This study
pGM59	Coding sequence of Zorya II *Klebsiella oxytoca* 571.31(G0D99_RS19745, G0D99_RS19740 and G0D99_RS19735) in pGM39	This study
pGM60	Coding sequence of Zorya II *Legionella lansingensis* NCTC12830 (CKV79_RS08030, CKV79_RS08025 and CKV79_RS08020) in pGM39	This study
pGM61	Coding sequence of Zorya II S. *marcescens* UMH9 (BVG96_RS22930, BVG96_RS22925 and BVG96_RS22920) in pGM39	This study
pGM62	Coding sequence of Zorya II *E. coli* KTE66 in pGM39	This study
pGM63	Coding sequence of Zorya II *Pseudomonas jinjuensis* NBRC10347 in pGM39	This study
pGM64	Coding sequence of Zorya II *S. marcescens* strain AS012322 in pGM39	This study
pGM65	Coding sequence of Zorya II *Shewanella bicestrii* strain JAB-1(CF168_RS11660, CF168_RS11655 and CF168_RS11650) in pGM39	This study
pGM94	Coding sequence of ZorCD from *S. marcescens* ATCC 274 cloned downstream of ZorAB II (from *S. marcescens* UMH9) in pGM39	This study

pGM98	Coding sequence of ZorAB I from *S. marcescens* ATCC 274 and ZorE from *S. marcescens* UMH9) in pGM39	This study
pGM143	Coding sequence of Zorya II *E.coli* ATCC 8739 in pGM39, with ZorA II carrying a A210P mutation	This study
pGM144	Coding sequence of Zorya II *E.coli* ATCC 8739 in pGM39, with ZorA II carrying a V237A mutation	This study
pGM145	Coding sequence of Zorya II *E.coli* ATCC 8739 in pGM39, with ZorA II carrying a Q244A mutation	This study
pGM146	Coding sequence of Zorya II *E.coli* ATCC 8739 in pGM39, with ZorA II carrying a I282A mutation	This study
pGM147	Coding sequence of Zorya II *E.coli* ATCC 8739 in pGM39, with ZorA II carrying a T309A mutation	This study
pGM148	Coding sequence of Zorya II *E.coli* ATCC 8739 in pGM39, with ZorA II carrying a L346A mutation	This study
pGM149	Coding sequence of Zorya II *E.coli* ATCC 8739 in pGM39, with ZorA II carrying a E366A mutation	This study
pGM150	Coding sequence of Zorya II *E.coli* ATCC 8739 in pGM39, with ZorA II carrying a E402A mutation	This study

pGM152	Coding sequence of Zorya II *E.coli* ATCC 8739 in pGM39, with ZorB II carrying a E139A mutation	This study
pGM153	Coding sequence of Zorya II *E.coli* ATCC 8739 in pGM39, with ZorB II carrying a H141A mutation	This study
pGM154	Coding sequence of Zorya II *E.coli* ATCC 8739 in pGM39, with ZorB II carrying a T142A mutation	This study
pGM155	Coding sequence of Zorya II *E.coli* ATCC 8739 in pGM39, with ZorB II carrying a S143A mutation	This study
pGM156	Coding sequence of Zorya II *E.coli* ATCC 8739 in pGM39, with ZorB II carrying a D145A mutation	This study
pGM157	Coding sequence of Zorya II *E.coli* ATCC 8739 in pGM39, with ZorB II carrying a K188A mutation	This study
pGM158	Coding sequence of Zorya II *E.coli* ATCC 8739 in pGM39, with ZorB II carrying a Y196A mutation	This study
pGM159	Coding sequence of Zorya II *E.coli* ATCC 8739 in pGM39, with ZorB II carrying a R215A mutation	This study
pGM160	Coding sequence of Zorya II *E.coli* ATCC 8739 in pGM39, with ZorB II carrying a I232A mutation	This study

pGM161	Coding sequence of Zorya I *S. marcescens* ATCC274 in pGM39, with ZorA I carrying a E267A mutation	This study
pGM162	Coding sequence of Zorya I *S. marcescens* ATCC274 in pGM39, with ZorA I carrying a A280P mutation	This study
pGM163	Coding sequence of Zorya I *S. marcescens* ATCC274in pGM39, with ZorA I carrying a S302P mutation	This study
pGM164	Coding sequence of Zorya I *S. marcescens* ATCC274 in pGM39, with ZorA I carrying a S319A mutation	This study
pGM165	Coding sequence of Zorya I *S. marcescens* ATCC274 in pGM39, with ZorA I carrying a L361A mutation	This study
pGM166	Coding sequence of Zorya I *S. marcescens* ATCC274 in pGM39, with ZorA I carrying a Q392A mutation	This study
pGM167	Coding sequence of Zorya I *S. marcescens* ATCC274in pGM39, with ZorA I carrying a L423A mutation	This study
pGM168	Coding sequence of Zorya I *S. marcescens* ATCC274in pGM39, with ZorA II carrying a K444A mutation	This study
pGM169	Coding sequence of Zorya I *S. marcescens* ATCC274in pGM39, with ZorA I carrying a E481A mutation	This study

pGM170	Coding sequence of Zorya I *S. marcescens* ATCC274 in pGM39, with ZorA I carrying a A520P mutation	This study
pGM171	Coding sequence of Zorya I *S. marcescens* ATCC274 in pGM39, with ZorA I carrying a S598A mutation	This study
pGM172	Coding sequence of Zorya I *S. marcescens* ATCC274in pGM39, with ZorA I carrying a Y631A mutation	This study
pGM173	Coding sequence of Zorya I *S. marcescens* ATCC274in pGM39, with ZorA I carrying a V664A mutation	This study
pGM176	Coding sequence of Zorya I *S. marcescens* ATCC274 in pGM39, with ZorB I carrying a H186A mutation	This study
pGM180	Coding sequence of Zorya I *S. marcescens* ATCC274in pGM39, with ZorB II carrying a L199A mutation	This study
pGM181	Coding sequence of Zorya I *S. marcescens* ATCC274 in pGM39, with ZorB II carrying a R203A mutation	This study
pGM183	Coding sequence of Zorya I *S. marcescens* ATCC274 in pGM39, with ZorB II carrying a R254A mutation	This study
pGM185	Coding sequence of Zorya I *S. marcescens* ATCC274 in pGM39, with ZorB II carrying a R259A mutation	This study
pGM29	Coding sequence of ZorE from *E. coli* ATCC 8739 in pT12-derived plasmid. Insert was cloned in pT12-ecMotAB to replace ecMotAB	This study

	and in frame with the TEV site and 2xStrepII tag	
pGM287	Coding sequence of ZorBI $_{165\text{-}287}$ from *S. marcescens* ATCC 274 in pT12-derived plasmid. Insert was cloned in pT12-ecMotAB to replace ecMotAB and in frame with the TEV site and 2xStrepII tag	This study
pGM288	Coding sequence of ZorBII$_{115\text{-}235}$ from *E. coli* ATCC 8739 in pT12-derived plasmid. Insert was cloned in pT12-ecMotAB to replace ecMotAB and in frame with the TEV site and 2xStrepII tag	This study
pGM301	Coding sequence of ZorBII$_{115\text{-}235}$ carrying the S143A point mutation. Plasmid derived from pGM288.	This study
pGM304	Coding sequence of ZorBII$_{115\text{-}235}$ carrying the R215A point mutation. Plasmid derived from pGM288.	This study
pGM306	Coding sequence of ZorBI $_{165\text{-}287}$ carrying the L199A point mutation. Plasmid derived from pGM287.	This study
pGM307	Coding sequence of ZorBI $_{165\text{-}287}$ carrying the H186A point mutation. Plasmid derived from pGM287.	This study
pGM309	Coding sequence of ZorBI $_{165\text{-}287}$ carrying the R254A point mutation. Plasmid derived from pGM287.	This study
pGM311	Coding sequence of ZorBI $_{165\text{-}287}$ carrying the R203A point mutation. Plasmid derived from pGM287.	This study

pGM312	Coding sequence of ZorBI $_{165\text{-}287}$ carrying the R259A point mutation. Plasmid derived from pGM287.	This study
pGM328	Coding sequence of ZorBII$_{115\text{-}235}$ carrying the H141A point mutation. Plasmid derived from pGM288.	This study
pGM329	Coding sequence of ZorBII$_{115\text{-}235}$ carrying the R230A point mutation. Plasmid derived from pGM288.	This study
pGM41	Coding sequence of ZorA, followed by ZorB fused to a TEV site and 2xStrepII tag and ZorE fused to a His6x tag in pT12. Sequences were amplified from *E. coli* ATCC 8739	This study
pGM289	Coding sequence ZorB fused to a TEV site and 2xStrepII tag followed by ZorE fused to a His6x tag in pT12. Sequences were amplified from *E. coli* ATCC 8739	This study
pGM290	Coding sequence ZorA followed by ZorE fused to a His6x tag in pT12. Sequences were amplified from *E. coli* ATCC 8739	This study
pGM291	Coding sequence of ZorE fused to a His6x tag in pT12. Sequences were amplified from *E. coli* ATCC 8739	This study
pGM67	Coding sequence of ZorB fused to a TEV site and 2xStrepII tag inserted in its native position in the Zorya II operon in pGM23.	This study
pSUPROM	Low copy plasmid carrying a constitutive Tat promoter and a Kanamycin resistance gene.	[3]
pGM196	Coding sequence of Zorya II from *E. coli* ATCC 8739, with its own promoter and terminator, cloned in pSUPROM. Cloning designed to	This study

	remove the Tat promoter from pSUPROM and replace with Zorya II own promoter.	
pGM336	pSUPROM derivative where Tat promoter has been replaced by Zorya II promoter. Promoter sequence obtained from *E. coli* ATCC 8739	This study
pGM337	Coding sequence of ZorBE in pGM336. . Sequences were amplified from *E. coli* ATCC 8739	This study
pGM338	Coding sequence of ZorAE in pGM336. . Sequences were amplified from *E. coli* ATCC 8739	This study
pGM339	Coding sequence of ZorE in pGM336. . Sequences were amplified from *E. coli* ATCC 8739	This study
pGM340	Coding sequence of ZorAB in pGM336. . Sequences were amplified from *E. coli* ATCC 8739	This study
pGM341	Coding sequence of ZorA, followed by ZorB fused to a TEV site and 2xStrepII tag and ZorE fused to a His6x tag in pGM336. Sequences were amplified from *E. coli* ATCC 8739	This study
pGM342	Coding sequence ZorB fused to a TEV site and 2xStrepII tag followed by ZorE fused to a His6x tag in pGM336. Sequences were amplified from *E. coli* ATCC 8739	This study
pGM343	Coding sequence of ZorA followed by ZorE fused to a His6x tag in pGM336. Sequences were amplified from *E. coli* ATCC 8739	This study

pGM344	Coding sequence of ZorE fused to a His6x tag in pGM336. Sequences were amplified from *E. coli* ATCC 8739	This study

1. Baumgarten, T. *et al.* Isolation and characterization of the E. coli membrane protein production strain Mutant56(DE3). *Sci. Rep.* **7**, 45089 (2017).
2. Deme, J. C. *et al.* Structures of the stator complex that drives rotation of the bacterial flagellum. *Nat. Microbiol.* **5**, 1553–1564 (2020).
3. Jack, R. L. *et al.* Coordinating assembly and export of complex bacterial proteins. *EMBO J.* **23**, 3962–3972 (2004).

Supplementary Table 2. Oligonucleotide primers and additional details for plasmid construction.

Plasmid	Sequence of relevant primers (5'-3')[a]	Description
pGM39	AACAGAAAAACCGACTAG	Forward primer delete ecMotAB-TEV-StrepII tag to generate empty vector by KLD
	GGTGAATTCCTCCTGAATTTCATTACG	Reverse primer delete ecMotAB-TEV-StrepII tag to generate empty vector by KLD
pGM15	GAAAACCTGTACTTCCAGGGTCAAT	Forward primer to clone ZorAB II from *Sulfuricurvum kujiense* DSM 16994 (SULKU_RS11880 and SULKU_RS11885) in pT12-derived plasmid in frame with a C-term Strep II tag by NEBuilder HiFi DNA Assembly. Primer is used to linearise vector
	GGTGAATTCCTCCTGAATTTCATTACGAC	Reverse primer to clone ZorAB II from *Sulfuricurvum kujiense* DSM 16994 (SULKU_RS11880 and SULKU_RS11885) in pT12-derived plasmid in frame with a C-term Strep II tag by NEBuilder HiFi DNA Assembly. Primer is used to linearise vector
	TTTTTAGACTGGTCGTAATGAAATTCAGGAGGAATTCACCGTGATTCATAATATGGCCTATTTCGGAGTCG	Forward primer to clone ZorAB II from *Sulfuricurvum kujiense* DSM 16994 (SULKU_RS11880 and SULKU_RS11885) in pT12-derived plasmid in frame with a C-term Strep II tag by NEBuilder HiFi DNA

		Assembly. Primer is used to amplify genes of interest
	GGCTCCAGCTCCCGAATTGACCCTGG AAGTACAGGTTTTCTTTTTCTACTATAT CGGCGACTTTACGTTCAATG	Reverse primer to clone ZorAB II from *Sulfuricurvum kujiense* DSM 16994 (SULKU_RS11880 and SULKU_RS11885) in pT12-derived plasmid in frame with a C-term Strep II tag by NEBuilder HiFi DNA Assembly. Primer is used to amplify genes of interest
pGM54	GAAAACCTGTACTTCCAGGGTCAAT	Forward primer to clone ZorAB I Shewanella sp. ANA-3 (SHEWANA3_RS19785 SHEWANA3_RS19790 and SHEWANA3_RS19795) in pT12-derived plasmid in frame with a C-term Strep II tag by NEBuilder HiFi DNA Assembly. Primer is used to linearise vector
	GGTGAATTCCTCCTGAATTTCATTACG AC	Reverse primer to clone ZorAB I Shewanella sp. ANA-3 (SHEWANA3_RS19785 SHEWANA3_RS19790 and SHEWANA3_RS19795) in pT12-derived plasmid in frame with a C-term Strep II tag by NEBuilder HiFi DNA Assembly. Primer is used to linearise vector
	AAATTCAGGAGGAATTCACCATGGCAA CAGAAAGACAAATTG	Forward primer to clone ZorAB I Shewanella sp. ANA-3 (SHEWANA3_RS19785 SHEWANA3_RS19790) in pT12-derived plasmid in frame with a C-

		term Strep II tag by NEBuilder HiFi DNA Assembly. Primer is used to amplify genes of interest
	CCCTGGAAGTACAGGTTTTCAACATTCTTCGCTTTTTCG	Reverse primer to clone ZorAB I Shewanella sp. ANA-3 (SHEWANA3_RS19785 SHEWANA3_RS19790 and SHEWANA3_RS19795) in pT12-derived plasmid in frame with a C-term Strep II tag by NEBuilder HiFi DNA Assembly. Primer is used to amplify genes of interest
pGM99	AACATTCTTCGCTTTTTCG	Forward primer to clone ZorCD operon from Shewanella sp. ANA-3 in pGM54 by NEBuilder HiFi DNA Assembly, removing the Strep II tag. Primer is used to linearise vector
	AACAGAAAAACCGACTAG	Reverse primer to clone ZorCD operon from Shewanella sp. ANA-3 in pGM54 by NEBuilder HiFi DNA Assembly, removing the Strep II tag. Primer is used to linearise vector
	ACGAAAAAGCGAAGAATGTTTAAAGCATGTGCGTGGAG	Forward primer to clone ZorCD operon from Shewanella sp. ANA-3 in pGM54 by NEBuilder HiFi DNA Assembly, removing the Strep II tag. Primer is used to amplify genes of interest
	AGCTAGTCGGTTTTTCTGTTCTAACGAACTACATATCGTTG	Reverse primer to clone ZorCD operon from Shewanella sp. ANA-3 in pGM54 by NEBuilder HiFi DNA Assembly, removing the Strep II tag.

		Primer is used to amplify genes of interest
pGM23	GGTGAATTCCTCCTGAATTTCATTACG	Forward primer to clone Zorya II operon from *E. coli* ATCC 8739 (ECOLC_RS20900, ECOLC_RS20905 and ECOLC_RS20910) in pGM39 by NEBuilder HiFi DNA Assembly. Primer is used to linearise vector
	TAAAACAGAAAAACCGACTAGCTTGG	Reverse primer to clone Zorya II operon from *E. coli* ATCC 8739 (ECOLC_RS20900, ECOLC_RS20905 and ECOLC_RS20910) in pGM39 by NEBuilder HiFi DNA Assembly. Primer is used to linearise vector
	AAATTCAGGAGGAATTCACCATGTTAG CGCAGCTTTTTG	Forward primer to clone Zorya II operon from *E. coli* ATCC 8739 (ECOLC_RS20900, ECOLC_RS20905 and ECOLC_RS20910) in pGM39 by NEBuilder HiFi DNA Assembly. Primer is used to amplify genes of interest
	AGGATCCCCGGGTACCCTAGTTGAGA CTCGCCAACCATGGGC	Reverse primer to clone Zorya II operon from *E. coli* ATCC 8739 (ECOLC_RS20900, ECOLC_RS20905 and ECOLC_RS20910) in pGM39 by NEBuilder HiFi DNA Assembly. Primer is used to amplify genes of interest

pGM35	TCGATCATGGATAAGATTATAG	Forward primer delete ZorA from pGM23 by KLD
	GGTGAATTCCTCCTGAATTTCATTACG	Reverse primer delete ZorA from pGM23 by KLD
pGM36	GAGATGAAATTATCTATC	Forward primer delete ZorB from pGM23 by KLD
	GATCGATTACCCTCGATG	Reverse primer delete ZorB from pGM23 by KLD
pGM37	GAGATGAAATTATCTATC	Forward primer delete ZorAB from pGM23 by KLD
	GGTGAATTCCTCCTGAATTTCATTACG	Reverse primer delete ZorAB from pGM23 by KLD
pGM38	AACAGAAAAACCGACTAG	Forward primer delete ZorE from pGM23 by KLD
	GATTATTCAGGAGTAAGAG	Reverse primer delete ZorE from pGM23 by KLD
pGM46	AACCTAATGGCAGGGCTGATGATG	Forward primer introduce a D24N mutation in ZorB in pGM23 by KLD
	TGACATGGATACCCAATGTTC	Reverse primer introduce a D24N mutation in ZorB in pGM23 by KLD
pGM55	AAATAAAATGGCAAAAAAC	Forward primer to delete a aa106-550 in ZorA in pGM23 by KLD
	TAATCGATCATGGATAAGATTATAG	Reverse primer to delete a aa106-550 in ZorA in pGM23 by KLD
pGM70	GGTGAATTCCTCCTGAATTTCATTACG	Forward primer to clone Zorya I from *S. marcescens* ATCC 274(SMATCC274_RS21230, SMATCC274_RS21235 SMATCC274_RS21240 and SMATCC274_RS21245 in pGM39 by NEBuilder HiFi DNA Assembly. Primer is used to linearise vector

	TAAAACAGAAAAACCGACTAGCTTGG	Reverse primer to clone Zorya I from *S. marcescens* ATCC 274(SMATCC274_RS21230, SMATCC274_RS21235 SMATCC274_RS21240 and SMATCC274_RS21245 in pGM39 by NEBuilder HiFi DNA Assembly. Primer is used to linearise vector
	AAATTCAGGAGGAATTCACCATGGATTCGCTGCTGCTTC	Forward primer to clone Zorya I from *S. marcescens* ATCC 274(SMATCC274_RS21230, SMATCC274_RS21235 SMATCC274_RS21240 and SMATCC274_RS21245 in pGM39 by NEBuilder HiFi DNA Assembly. Primer is used to amplify genes of interest
	AGCTAGTCGGTTTTTCTGTTCTAACTGATTCGATATGAACC	Reverse primer to clone Zorya I from *S. marcescens* ATCC 274(SMATCC274_RS21230, SMATCC274_RS21235 SMATCC274_RS21240 and SMATCC274_RS21245 in pGM39 by NEBuilder HiFi DNA Assembly. Primer is used to amplify genes of interest
pGM85	GGTGAATTCCTCCTGAATTTCATTAC	Forward primer delete ZorAB from pGM70 by KLD
	ATGAAATTAAGCGATATC	Reverse primer delete ZorAB from pGM70 by KLD
pGM86	TCATAGCGGCCTCTTCTTCAG	Forward primer delete ZorAB from pGM70 by KLD

	AACAGAAAAACCGACTAGCTTGGCTG	Reverse primer delete ZorAB from pGM70 by KLD
pGM124	GGTGAATTCCTCCTGAATTTCATTACG	Forward primer to clone ZorABC from *S. marcescens* ATCC 274 in pGM39 by NEBuilder HiFi DNA Assembly. Primer is used to linearise vector
	TAAAACAGAAAAACCGACTAGCTTGG	Reverse primer to clone ZorABC from *S. marcescens* ATCC 274 in pGM39 by NEBuilder HiFi DNA Assembly. Primer is used to linearise vector
	AAATTCAGGAGGAATTCACCATGGATTCGCTGCTGCTTC	Forward primer to clone ZorABC from *S. marcescens* ATCC 274 in pGM39 by NEBuilder HiFi DNA Assembly. Primer is used to amplify genes of interest
	AGCTAGTCGGTTTTTCTGTTTCATGACAGATACTCAATTTGTTCAC	Reverse primer to clone ZorABC from *S. marcescens* ATCC 274 in pGM39 by NEBuilder HiFi DNA Assembly. Primer is used to amplify genes of interest
pGM125	GGTGAATTCCTCCTGAATTTCATTACG	Forward primer to clone ZorBCD from *S. marcescens* ATCC 274 in pGM39 by NEBuilder HiFi DNA Assembly. Primer is used to linearise vector
	TAAAACAGAAAAACCGACTAGCTTGG	Reverse primer to clone ZorBCD from *S. marcescens* ATCC 274 in pGM39 by NEBuilder HiFi DNA Assembly. Primer is used to linearise vector

	AAATTCAGGAGGAATTCACCATGAGAGCCAACGCCAGTC	Forward primer to clone ZorBCD from *S. marcescens* ATCC 274 in pGM39 by NEBuilder HiFi DNA Assembly. Primer is used to amplify genes of interest
	AGCTAGTCGGTTTTTCTGTTCTAACTGATTCGATATGAACCGTCATC	Reverse primer to clone ZorBCD from *S. marcescens* ATCC 274 in pGM39 by NEBuilder HiFi DNA Assembly. Primer is used to amplify genes of interest
pGM140	AATATGACGGTCAGCTTTCTTTTTATTGTTATG	Forward primer to introduce aD24N mutation in ZorB in pGM70 by NEBuilder HiFi DNA Assembly.
	GAAAGCTGACCGTCATATTTGTCATTGAGACAAAAAC	Reverse primer to introduce aD24N mutation in ZorB in pGM70 by NEBuilder HiFi DNA Assembly.
pGM141	TCATAGCGGCCTCTTCTTC	Forward primer to clone ZorD from *S. marcescens* ATCC 274 downstream of ZorAB I in pGM86 by NEBuilder HiFi DNA Assembly. Primer is used to linearise vector
	AACAGAAAAACCGACTAGCTTGGCTG	Reverse primer to clone ZorD from *S. marcescens* ATCC 274 downstream of ZorAB I in pGM86 by NEBuilder HiFi DNA Assembly. Primer is used to linearise vector
	tgaagaagaggccgctatgaATGACATTTGCTTTCAATCATAATG	Forward primer to clone ZorD from *S. marcescens* ATCC 274 downstream of ZorAB I in pGM86 by NEBuilder HiFi DNA Assembly. Primer is used to amplify genes of interest

	agctagtcggtttttctgttCTAACTGATTCGATATGAACC	Reverse primer to clone ZorD from *S. marcescens* ATCC 274 downstream of ZorAB I in pGM86 by NEBuilder HiFi DNA Assembly. Primer is used to amplify genes of interest
pGM142	ATGAAATTAAGCGATATCTTCC	Forward primer to clone ZorA I from *S. marcescens* ATCC 274 upstream of ZorCD in pGM85 by NEBuilder HiFi DNA Assembly. Primer is used to linearise vector
	GGTGAATTCCTCCTGAATTTCATTACG	Reverse primer to clone ZorA I from *S. marcescens* ATCC 274 upstream of ZorCD in pGM85 by NEBuilder HiFi DNA Assembly. Primer is used to linearise vector
	AAATTCAGGAGGAATTCACCATGGATTCGCTGCTGCTTC	Forward primer to clone ZorA I from *S. marcescens* ATCC 274 upstream of ZorCD in pGM85 by NEBuilder HiFi DNA Assembly. Primer is used to amplify genes of interest
	AAGATATCGCTTAATTTCATTCATGAGATTCGCCCTTG	Reverse primer to clone ZorA I from *S. marcescens* ATCC 274 upstream of ZorCD in pGM85 by NEBuilder HiFi DNA Assembly. Primer is used to amplify genes of interest
pGM223	AATAGAAAGATCTTCGAGGCTG	Forward primer to delete a aa237-678 in ZorA in pGM70 by KLD
	ATGAGAGCCAACGCCAGTC	Reverse primer to delete a aa237-678 in ZorA in pGM70 by KLD
pGM224	GCTGGCAAAAAAAGCGAGC	Forward primer to delete a aa43-287 in ZorB in pGM70 by KLD

	ATGAAATTAAGCGATATC	Reverse primer to delete a aa43-287 in ZorB in pGM70 by KLD
pGM59	GGTGAATTCCTCCTGAATTTCATTACG	Forward primer to clone Zorya II operon from *Klebsiella oxytoca* 571.31(G0D99_RS19745, G0D99_RS19740 and G0D99_RS19735) in pGM39 by NEBuilder HiFi DNA Assembly. Primer is used to linearise vector
	TAAAACAGAAAAACCGACTAGCTTGG	Reverse primer to clone Zorya II operon from from *Klebsiella oxytoca* 571.31 in pGM39 by NEBuilder HiFi DNA Assembly. Primer is used to linearise vector
	TTTTTAGACTGGTCGTAATGAAATTCAG GAGGAATTCACCATGAAGAAAAGACTT CTACTTCTGTTGTTACTCAT	Forward primer to clone Zorya II operon from *Klebsiella oxytoca* 571.31 in pGM39 by NEBuilder HiFi DNA Assembly. Primer is used to amplify genes of interest
	AGCTAGTCGGTTTTTCTGTTTCAGCAC TTGTAATTTGTC	Reverse primer to clone Zorya II operon from *Klebsiella oxytoca* 571.31in pGM39 by NEBuilder HiFi DNA Assembly. Primer is used to amplify genes of interest
pGM60	GGTGAATTCCTCCTGAATTTCATTACG	Forward primer to clone Zorya II operon II *Legionella lansingensis* NCTC12830 (CKV79_RS08030, CKV79_RS08025 and CKV79_RS08020) in pGM39 by NEBuilder HiFi DNA Assembly. Primer is used to linearise vector
	TAAAACAGAAAAACCGACTAGCTTGG	Reverse primer to clone Zorya II operon from *Legionella lansingensis*

		NCTC12830 in pGM39 by NEBuilder HiFi DNA Assembly. Primer is used to linearise vector
	TTTTTAGACTGGTCGTAATGAAATTCAGGAGGAATTCACCATGAATAAAATATTTGCTTTATTGTTAGCCCTAGTTTCC	Forward primer to clone Zorya II operon from *Legionella lansingensis* NCTC12830 in pGM39 by NEBuilder HiFi DNA Assembly. Primer is used to amplify genes of interest
	AGCTAGTCGGTTTTTCTGTTTTAAAATTTATTCTTTTTTATTTGCGC	Reverse primer to clone Zorya II operon from *Legionella lansingensis* NCTC12830 in pGM39 by NEBuilder HiFi DNA Assembly. Primer is used to amplify genes of interest
pGM61	GGTGAATTCCTCCTGAATTTCATTACG	Forward primer to clone Zorya II operon II from S. *marcescens* UMH9 (BVG96_RS22930, BVG96_RS22925 and BVG96_RS22920) in pGM39 by NEBuilder HiFi DNA Assembly. Primer is used to linearise vector
	TAAAACAGAAAAACCGACTAGCTTGG	Reverse primer to clone Zorya II operon from S. *marcescens* UMH9 in pGM39 by NEBuilder HiFi DNA Assembly. Primer is used to linearise vector
	TTTTTAGACTGGTCGTAATGAAATTCAGGAGGAATTCACCATGTTAGCGCAACTTTTTGAACATCTG	Forward primer to clone Zorya II operon from S. *marcescens* UMH9 in pGM39 by NEBuilder HiFi DNA Assembly. Primer is used to amplify genes of interest
	AGCTAGTCGGTTTTTCTGTTTCAGAAAACAACTCCGGAC	Reverse primer to clone Zorya II operon from S. *marcescens* UMH9 in pGM39 by NEBuilder HiFi DNA

		Assembly. Primer is used to amplify genes of interest
pGM62	GGTGAATTCCTCCTGAATTTCATTACG	Forward primer to clone Zorya II operon II from *E. coli* KTE66 in pGM39 by NEBuilder HiFi DNA Assembly. Primer is used to linearise vector
	TAAAACAGAAAAACCGACTAGCTTGG	Reverse primer to clone Zorya II operon from *E. coli* KTE66 in pGM39 by NEBuilder HiFi DNA Assembly. Primer is used to linearise vector
	AAATTCAGGAGGAATTCACCATGCTGG CACAGCTTTTTG	Forward primer to clone Zorya II operon from *E. coli* KTE66 in pGM39 by NEBuilder HiFi DNA Assembly. Primer is used to amplify genes of interest
	AGCTAGTCGGTTTTTCTGTTCTACACC GCGGTGAAGATG	Reverse primer to clone Zorya II operon from *E. coli* KTE66 in pGM39 by NEBuilder HiFi DNA Assembly. Primer is used to amplify genes of interest
pGM63	GGTGAATTCCTCCTGAATTTCATTACG	Forward primer to clone Zorya II operon II from *Pseudomonas jinjuensis* NBRC10347 in pGM39 by NEBuilder HiFi DNA Assembly. Primer is used to linearise vector
	TAAAACAGAAAAACCGACTAGCTTGG	Reverse primer to clone Zorya II operon from *Pseudomonas jinjuensis* NBRC10347 in pGM39 by NEBuilder HiFi DNA Assembly. Primer is used to linearise vector

	AAATTCAGGAGGAATTCACCATGATTT CCGAGCGCCTG	Forward primer to clone Zorya II operon from *Pseudomonas jinjuensis* NBRC10347 in pGM39 by NEBuilder HiFi DNA Assembly. Primer is used to amplify genes of interest
	AGCTAGTCGGTTTTTCTGTTTCAACCA AGACCGCCACAAATTTC	Reverse primer to clone Zorya II operon from *Pseudomonas jinjuensis* NBRC10347 in pGM39 by NEBuilder HiFi DNA Assembly. Primer is used to amplify genes of interest
pGM64	GGTGAATTCCTCCTGAATTTCATTACG	Forward primer to clone Zorya II operon II from II *S. marcescens* strain AS012322 in pGM39 by NEBuilder HiFi DNA Assembly. Primer is used to linearise vector
	TAAAACAGAAAAACCGACTAGCTTGG	Reverse primer to clone Zorya II operon from II *S. marcescens* strain AS012322 in pGM39 by NEBuilder HiFi DNA Assembly. Primer is used to linearise vector
	AAATTCAGGAGGAATTCACCATGTTAG CGCAACTTTTTG	Forward primer to clone Zorya II operon from II *S. marcescens* strain AS012322 in pGM39 by NEBuilder HiFi DNA Assembly. Primer is used to amplify genes of interest
	CAAGCTAGTCGGTTTTTCTGTCAGAAA ACAACTCCGGAC	Reverse primer to clone Zorya II operon from II *S. marcescens* strain AS012322 in pGM39 by NEBuilder HiFi DNA Assembly. Primer is used to amplify genes of interest
pGM94	TTACTCCTGAATAATCTTTCTAATC	Forward primer to clone ZorCD downstream of ZorAB II in pGM61 by

		NEBuilder HiFi DNA Assembly. Primer is used to linearise vector
	TAAAACAGAAAAACCGACTAGCTTGGC	Reverse primer to clone ZorCD downstream of ZorAB II in pGM61 by NEBuilder HiFi DNA Assembly. Primer is used to linearise vector
	GTCGCATAATTCAGGAATAGATGAAATTAAGCGATATCTTCC	Forward primer to clone ZorCD downstream of ZorAB II in pGM61 by NEBuilder HiFi DNA Assembly Primer is used to amplify genes of interest
	GCCAAGCTAGTCGGTTTTTCTGTTTTACTAACTGATTCGATATGAACC	Reverse primer to clone ZorCD downstream of ZorAB II in pGM61 by NEBuilder HiFi DNA Assembly.. Primer is used to amplify genes of interest
pGM98	GGTGAATTCCTCCTGAATTTCATTACG	Forward primer to clone ZorE from pGM61 downstream of ZorAB I in pGM70 by NEBuilder HiFi DNA Assembly. Primer is used to linearise vector
	AACAGAAAAACCGACTAGCTTGGCTG	Reverse primer to clone ZorE from pGM61 downstream of ZorAB I in pGM70 by NEBuilder HiFi DNA Assembly. Primer is used to linearise vector
	TGAAGAAGAGGCCGCTATGAATGAAGCTAACTGTTGATTTTTC	Forward primer to clone ZorE from pGM61 downstream of ZorAB I in pGM70 by NEBuilder HiFi DNA Assembly Primer is used to amplify genes of interest
	AACAGAAAAACCGACTAGCTTGTCAGAAAACAACTCCGGAC	Reverse primer to ZorE from pGM61 downstream of ZorAB I in pGM70 by

		NEBuilder HiFi DNA Assembly.. Primer is used to amplify genes of interest
pGM143	CCTTTGCGACAAGTCATTATTGATTTTAATG	Forward primer to introduce a A210P mutation in ZorA II in pGM23 by NEBuilder HiFi DNA Assembly.
	CTTGTCGCAAAGGATCAATAATTTGTTCTG	Reverse primer to introduce A210P mutation in ZorA in pGM23 by NEBuilder HiFi DNA Assembly.
pGM144	GCAAAAAAACTTGTTGAGTGGCAGGGAAATTATAAAAC	Forward primer to introduce a V237A mutation in ZorA II in pGM23 by NEBuilder HiFi DNA Assembly.
	CAAGTTTTTTTGCAGAGGCATCAAGAGCTTTAAAG	Reverse primer to introduce V237A mutation in ZorA II in pGM23 by NEBuilder HiFi DNA Assembly.
pGM145	GCGGGAAATTATAAAACGCAAATTGAGCAG	Forward primer to introduce a Q244A mutation in ZorA II in pGM23 by NEBuilder HiFi DNA Assembly.
	GTTTTATAATTTCCCGCCCACTCAACAAGTTTTTTTAC	Reverse primer to introduce Q244A mutation in ZorA II in pGM23 by NEBuilder HiFi DNA Assembly.
pGM146	GCTCCTCTGGCTATGTCTGAACTGCGTGAAG	Forward primer to introduce a I282A mutation in ZorA II in pGM23 by NEBuilder HiFi DNA Assembly.
	CATAGCCAGAGGAGCTTCTTTACATTCTTC	Reverse primer to introduce I282A mutation in ZorA II in pGM23 by NEBuilder HiFi DNA Assembly.
pGM147	GCCTTTGTCGCCATCCGCGATAAAGCTACAAC	Forward primer to introduce a T309A mutation in ZorA II in pGM23 by NEBuilder HiFi DNA Assembly.

	GATGGCGACAAAGGCTTCTAAATGGC GGGAG	Reverse primer to introduce T309A mutation in ZorA II in pGM23 by NEBuilder HiFi DNA Assembly.
pGM148	GCTGAGCAAACCAGCCAGCAAATACTT CTTAATG	Forward primer to introduce a L346A mutation in ZorA II in pGM23 by NEBuilder HiFi DNA Assembly.
	CTGGTTTGCTCAGCAGATGCACTAACA TTTG	Reverse primer to introduce L346A mutation in ZorA II in pGM23 by NEBuilder HiFi DNA Assembly.
pGM149	GCAGGTACCGAAGGATTCAGACAATC GGTTAC	Forward primer to introduce a E366A mutation in ZorA II in pGM23 by NEBuilder HiFi DNA Assembly.
	GAATCCTTCGGTACCTGCATCCAGGG CAAC	Reverse primer to introduce E366A mutation in ZorA II in pGM23 by NEBuilder HiFi DNA Assembly.
pGM150	GCAACAATTACTGAAATGAAACAAAGT GGTGAAG	Forward primer to introduce a E402A mutation in ZorA II in pGM23 by NEBuilder HiFi DNA Assembly.
	CATTTCAGTAATTGTTGCACCTAACGT GCTGGT	Reverse primer to introduce E402A mutation in ZorA II in pGM23 by NEBuilder HiFi DNA Assembly.
pGM152	GCAGGTCACACAAGTACTGACTGGAC AGGAACAACGAATC	Forward primer to introduce a E139A mutation in ZorB II in pGM23 by NEBuilder HiFi DNA Assembly.
	AGTACTTGTGTGACCTGCAATGCGGAC TTCAGTAA	Reverse primer to introduce E139A mutation in ZorB II in pGM23 by NEBuilder HiFi DNA Assembly.
pGM153	GCCACAAGTACTGACTGGACAGGAAC AACGAATC	Forward primer to introduce a H141A mutation in ZorB II in pGM23 by NEBuilder HiFi DNA Assembly.

	GTCAGTACTTGTGGCACCTTCAATGCG GACTTC	Reverse primer to introduce H141A mutation in ZorB II in pGM23 by NEBuilder HiFi DNA Assembly.
pGM154	GCAAGTACTGACTGGACAGGAACAAC GAATCCTG	Forward primer to introduce a T142A mutation in ZorB II in pGM23 by NEBuilder HiFi DNA Assembly.
	GTCAGTACTTGCGTGACCTTCAATGCG GACTTCAG	Reverse primer to introduce T142A mutation in ZorB II in pGM23 by NEBuilder HiFi DNA Assembly.
pGM155	GCTACTGACTGGACAGGAACAACGAAT CCTG	Forward primer to introduce a S143A mutation in ZorB II in pGM23 by NEBuilder HiFi DNA Assembly.
	GTCAGTAGCTGTGTGACCTTCAATGCG GACTTC	Reverse primer to introduce S143A mutation in ZorB II in pGM23 by NEBuilder HiFi DNA Assembly.
pGM156	GCCTGGACAGGAACAACGAATCCTGA TATTG	Forward primer to introduce a D145A mutation in ZorB II in pGM23 by NEBuilder HiFi DNA Assembly.
	CTGTCCAGGCAGTACTTGTGTGACCTT CAATG	Reverse primer to introduce D145A mutation in ZorB II in pGM23 by NEBuilder HiFi DNA Assembly.
pGM157	GCAAGTAAATTTGCCGCAGTAGGTTAT TCATC	Forward primer to introduce a K188A mutation in ZorB II in pGM23 by NEBuilder HiFi DNA Assembly.
	CGGCAAATTTACTTGCAACCCATTGTT GGTGTGTC	Reverse primer to introduce K188A mutation in ZorB II in pGM23 by NEBuilder HiFi DNA Assembly.
pGM158	GCTTCATCTGCACATCCCATTCTTGAT AAAAC	Forward primer to introduce a Y196A mutation in ZorB II in pGM23 by NEBuilder HiFi DNA Assembly.
	GATGTGCAGATGAAGCACCTACTGCG GCAAAT	Reverse primer to introduce Y196A mutation in ZorB II in pGM23 by NEBuilder HiFi DNA Assembly.

pGM159	GCTCGTGTCACCTTCAAAGTTGTAACAAATG	Forward primer to introduce a R215A mutation in ZorB II in pGM23 by NEBuilder HiFi DNA Assembly.
	CTTTGAAGGTGACACGAGCAGAGCGATTAGG	Reverse primer to introduce R215A mutation in ZorB II in pGM23 by NEBuilder HiFi DNA Assembly.
pGM160	GCTATTCAGGAGTAAGAGATGAAATTATC	Forward primer to introduce a I232A mutation in ZorB II in pGM23 by NEBuilder HiFi DNA Assembly.
	CTTACTCCTGAATAGCCTTTCTAATCTGCAAC	Reverse primer to introduce I232A mutation in ZorB II in pGM23 by NEBuilder HiFi DNA Assembly.
pGM161	GCAGACCTTCCAAGAGCGATCTCTGAGTCTATTAG	Forward primer to introduce a E267A mutation in ZorA I in pGM70 by NEBuilder HiFi DNA Assembly.
	CTCTTGGAAGGTCTGCGCGCAACGGACGACCAATCTCAG	Reverse primer to introduce a E267A mutation in ZorA I in pGM70 by NEBuilder HiFi DNA Assembly.
pGM162	CCTATTACGCCGGTTATCGAACAAGTAAGCAGGTTG	Forward primer to introduce a A280P mutation in ZorA I in pGM70 by NEBuilder HiFi DNA Assembly.
	CGATAACCGGCGTAATAGGCGTACTAATAGACTCAGAGATC	Reverse primer to introduce a A280P mutation in ZorA I in pGM70 by NEBuilder HiFi DNA Assembly.
pGM163	GGCCTCTCTTCACGTTTTTCTGAAGATGTTGGTCG	Forward primer to introduce a S302P mutation in ZorA I in pGM70 by NEBuilder HiFi DNA Assembly.
	GTGAAGAGAGGCCTTGTACCATCTCTCCCACACCATC	Reverse primer to introduce a S302P mutation in ZorA I in pGM70 by NEBuilder HiFi DNA Assembly.
pGM164	GGCCTCTCTTCACGTTTTTCTGAAGATGTTGGTCG	Forward primer to introduce a S319A mutation in ZorA I in pGM70 by NEBuilder HiFi DNA Assembly.

	GTGAAGAGAGGCCTTGTACCATCTCTCCCACACCATC	Reverse primer to introduce a S319A mutation in ZorA I in pGM70 by NEBuilder HiFi DNA Assembly.
pGM165	GGCGAACGTATTTCCCTGGCAGGAGACCAGATTAAATTG	Forward primer to introduce a L361A mutation in ZorA I in pGM70 by NEBuilder HiFi DNA Assembly.
	CAGGGAAATACGTTCGCCGGCTTGTGCCAGAGCCCGAC	Reverse primer to introduce a L361A mutation in ZorA I in pGM70 by NEBuilder HiFi DNA Assembly.
pGM166	GCGCGTGATGGTATGCAGCAAACGGCAGATACAGCAACATC	Forward primer to introduce a Q392A mutation in ZorA I in pGM70 by NEBuilder HiFi DNA Assembly.
	CTGCATACCATCACGCGCTGTTTCCACCGCCTGGCCTAAG	Reverse primer to introduce a Q392A mutation in ZorA I in pGM70 by NEBuilder HiFi DNA Assembly.
pGM167	GCAGGCATCAAAGACAATACCGGTGAGGGTGCGCGAG	Forward primer to introduce a L423A mutation in ZorA I in pGM70 by NEBuilder HiFi DNA Assembly.
	TATTGTCTTTGATGCCTGCCAGGGTTGAATTCATGGTCG	Reverse primer to introduce a L423A mutation in ZorA I in pGM70 by NEBuilder HiFi DNA Assembly.
pGM168	GCCGAAGTTGCAGCGAAACAGGGGGCTGAAGCAGCGCAG	Forward primer to introduce a K444A mutation in ZorA I in pGM70 by NEBuilder HiFi DNA Assembly.
	GTTTCGCTGCAACTTCGGCTTCGGTACGAATACCCAGAG	Reverse primer to introduce a K444A mutation in ZorA I in pGM70 by NEBuilder HiFi DNA Assembly.
pGM169	GCGGCCAGCGACGCGATTGGCCAGGCCGCAACACAAGTG	Forward primer to introduce a E481A mutation in ZorA I in pGM70 by NEBuilder HiFi DNA Assembly.
	CGTCGCTGGCCGCAGACGCTGATTCAGACATCCGCGCCTGC	Reverse primer to introduce a E481A mutation in ZorA I in pGM70 by NEBuilder HiFi DNA Assembly.

pGM170	GCATTGCTTTCTCCTCTTTCTGTTATCGCTGAAAAACTG	Forward primer to introduce a A520P mutation in ZorA I in pGM70 by NEBuilder HiFi DNA Assembly.
	GAGGAGAAAGCAATGCGCTGCTGGCTTTCTGGGTCATC	Reverse primer to introduce a A520P mutation in ZorA I in pGM70 by NEBuilder HiFi DNA Assembly.
pGM171	CCCTCGGAGAACGCTTCAGGAAGCTTCCGCACATC	Forward primer to introduce a S598A mutation in ZorA I in pGM70 by NEBuilder HiFi DNA Assembly.
	GCGTTCTCCGAGGGGACAGCACCTTCTTTAATCCCATC	Reverse primer to introduce a S598A mutation in ZorA I in pGM70 by NEBuilder HiFi DNA Assembly.
pGM172	GCTACGCTAAATAATCTTCAGTCTTTGATTGCGGCTTTAC	Forward primer to introduce a Y631A mutation in ZorA I in pGM70 by NEBuilder HiFi DNA Assembly.
	GATTATTTAGCGTAGCTTCAACCGCCCGATGCTGCCCG	Reverse primer to introduce a Y631A mutation in ZorA I in pGM70 by NEBuilder HiFi DNA Assembly.
pGM173	GCTCAACAGCAAGTTGCTACGTCGGTTGAAACGCTCTTC	Forward primer to introduce a V664A mutation in ZorA I in pGM70 by NEBuilder HiFi DNA Assembly.
	CAACTTGCTGTTGAGCACTGTCGAATGCCTTGCCAAG	Reverse primer to introduce a V664A mutation in ZorA I in pGM70 by NEBuilder HiFi DNA Assembly.
pGM175	GCAGGTCACACCGACTCCAAAGGCGAAGATGTTTATAAC	Forward primer to introduce a N184A mutation in ZorB I in pGM70 by NEBuilder HiFi DNA Assembly.
	GAGTCGGTGTGACCTGCAATCTGTACCGCTTCAATAATGG	Reverse primer to introduce a N184A mutation in ZorB I in pGM70 by NEBuilder HiFi DNA Assembly.
pGM176	GCCACCGACTCCAAAGGCGAAGATGTTTATAACCTGAATC	Forward primer to introduce a H186A mutation in ZorB I in pGM70 by NEBuilder HiFi DNA Assembly.

	CTTTGGAGTCGGTGGCACCTTCAATCTGTACCGCTTCAATAATG	Reverse primer to introduce a H186A mutation in ZorB I in pGM70 by NEBuilder HiFi DNA Assembly.
pGM180	GCTTCGACTAACCGAGCCATCACCACGTTTACCAGTATG	Forward primer to introduce a L199A mutation in ZorB I in pGM70 by NEBuilder HiFi DNA Assembly.
	GATGGCTCGGTTAGTCGAAGCATTCAGGTTATAAAC	Reverse primer to introduce a L199A mutation in ZorB I in pGM70 by NEBuilder HiFi DNA Assembly.
pGM181	GCAGCCATCACCACGTTTACCAGTATGCTTGCCGCAG	Forward primer to introduce a R203A mutation in ZorB I in pGM70 by NEBuilder HiFi DNA Assembly.
	GTAAACGTGGTGATGGCTGCGTTAGTCGAAAGATTC	Reverse primer to introduce a R203A mutation in ZorB I in pGM70 by NEBuilder HiFi DNA Assembly.
pGM183	GCCCGTATCGATCTCCGCATCATTATGCATACCCCGGC	Forward primer to introduce a R254A mutation in ZorB I in pGM70 by NEBuilder HiFi DNA Assembly.
	GAGATCGATACGGGCATTCATCGCCATATTTTGCGGTG	Reverse primer to introduce a R254A mutation in ZorB I in pGM70 by NEBuilder HiFi DNA Assembly.
pGM185	GCCATCATTATGCATACCCCGGCCAATGCAGCGGAAATTGAAG	Forward primer to introduce a R259A mutation in ZorB I in pGM70 by NEBuilder HiFi DNA Assembly.
	GTATGCATAATGATGGCGAGATCGATACGGCGATTCATC	Reverse primer to introduce a R259A mutation in ZorB I in pGM70 by NEBuilder HiFi DNA Assembly.
pGM29	GGTGAATTCCTCCTGAATTTCATTAC	Forward primer to clone ZorE from *E. coli* ATCC8739 in pT12-derived plasmid in frame with a C-term Strep II tag by NEBuilder HiFi DNA Assembly. Primer is used to linearise vector

	GAAAACCTGTACTTCCAGGGTC	Reverse primer to clone ZorE from *E. coli* ATCC8739 in pT12-derived plasmid in frame with a C-term Strep II tag by NEBuilder HiFi DNA Assembly. Primer is used to linearise vector
	AAATTCAGGAGGAATTCACCATGAAATTATCTATCGACATTTCAG	Forward primer to clone ZorE from *E. coli* ATCC8739 in pT12-derived plasmid in frame with a C-term Strep II tag by NEBuilder HiFi DNA Assembly. Primer is used to amplify genes of interest
	CCCTGGAAGTACAGGTTTTCCAATTTTGCTGGCGTAAAG	Reverse primer to clone ZorE from *E. coli* ATCC8739 in pT12-derived plasmid in frame with a C-term Strep II tag by NEBuilder HiFi DNA Assembly. Primer is used to amplify genes of interest
pGM288	GAAAACCTGTACTTCCAGGGTCAAT	Forward primer to clone ZorB $II_{115-235}$ from *E. coli* ATCC8739 in pT12-derived plasmid in frame with a C-term Strep II tag by NEBuilder HiFi DNA Assembly. Primer is used to linearise vector
	GGTGAATTCCTCCTGAATTTCATTACGAC	Reverse primer to clone ZorB $II_{115-235}$ from *E. coli* ATCC8739 in pT12-derived plasmid in frame with a C-term Strep II tag by NEBuilder HiFi DNA Assembly. Primer is used to linearise vector
	AAATTCAGGAGGAATTCACCCTTGACGACTTCTTTCCTC	Forward primer to clone ZorB $II_{115-235}$ from *E. coli* ATCC8739 in pT12-derived plasmid in frame with a C-

		term Strep II tag by NEBuilder HiFi DNA Assembly. Primer is used to amplify genes of interest. Primers also used to amplify correspondent point mutations.
	CCCTGGAAGTACAGGTTTTCCTCCTGAATAATCTTTC	Reverse primer to clone ZorB $II_{115\text{-}235}$ from *E. coli* ATCC8739 in pT12-derived plasmid in frame with a C-term Strep II tag by NEBuilder HiFi DNA Assembly. Primer is used to amplify genes of interest. Primers also used to amplify correspondent point mutations.
pGM287	GAAAACCTGTACTTCCAGGGTCAAT	Forward primer to clone ZorB $I_{165\text{-}287}$ from *S. marcescens* ATCC274 in pT12-derived plasmid in frame with a C-term Strep II tag by NEBuilder HiFi DNA Assembly. Primer is used to linearise vector
	GGTGAATTCCTCCTGAATTTCATTACGAC	Reverse primer to clone ZorB $I_{165\text{-}287}$ from *S. marcescens* ATCC274 in pT12-derived plasmid in frame with a C-term Strep II tag by NEBuilder HiFi DNA Assembly. Primer is used to linearise vector
	AAATTCAGGAGGAATTCACCGTCGGGTGGGATAAAAAC	Forward primer to clone ZorB $I_{165\text{-}287}$ from *S. marcescens* ATCC274 in pT12-derived plasmid in frame with a C-term Strep II tag by NEBuilder HiFi DNA Assembly. Primer is used to amplify genes of interest. Primers also used to amplify correspondent point mutations.

	CCCTGGAAGTACAGGTTTTCTAGCGGCCTCTTCTTCAGGG	Reverse primer to clone ZorB $I_{165\text{-}287}$ from *S. marcescens* ATCC274 in pT12-derived plasmid in frame with a C-term Strep II tag by NEBuilder HiFi DNA Assembly. Primer is used to amplify genes of interest. Primers also used to amplify correspondent point mutations.
pGM196	AGAATTCACTGGCCGTCGTTTTAC	Forward primer to clone Zorya II with its own promoter from *E. coli* ATCC8739 in pSUPROM by NEBuilder HiFi DNA Assembly. Primer binds to the vector and are designed to remove the Tat promoter to insert Zorya II with its own promoter and terminator
	TCTAGAGTCGACCCCTCG	Reverse primer to clone Zorya II with its own promoter from *E. coli* ATCC8739 in pSUPROM by NEBuilder HiFi DNA Assembly. Primer binds to the vector and are designed to remove the Tat promoter to insert Zorya II with its own
	AACGACGGCCAGTGAATTCTCCTGCCTTCCTTTGATAC	Forward primer to clone Zorya II with its own promoter from *E. coli* ATCC8739 in pSUPROM by NEBuilder HiFi DNA Assembly. Primer binds to insert.
	CTCGAGGGGTCGACTCTAGATTACAATTTTGCTGGCGTAAAG	Reverse primer to clone Zorya II with its own promoter from *E. coli* ATCC8739 in pSUPROM by

		NEBuilder HiFi DNA Assembly. Primer binds to insert.
pGM336	TCTAGAGTCGACCCCTCG	Forward primer to delete Zorya II from pSUPROM by KLD leaving Zorya II specific promoter in the to replace Tat promoter.
	TTTGATACCTGTGTAAATAATGG	Reverse primer to delete Zorya II from pSUPROM by KLD leaving ZoryaII specific promoter in the to replace Tat promoter.
pGM337	GATCGATTACCCTCGATG	Forward primer to delete ZorA from pGM196 by KLD.
	GATTATTCAGGAGTAAGAG	Reverse primer to delete ZorA from pGM196 by KLD.
pGM338	ATGGATAAGATTATAGGGAAAC	Forward primer to delete ZorB from pGM196 by KLD.
	GATCGATTACCCTCGATG	Reverse primer to delete ZorB from pGM196 by KLD.
pGM339	ATGGATAAGATTATAGGGAAAC	Forward primer to delete ZorAB from pGM196 by KLD.
	TTTGATACCTGTGTAAATAATGG	Reverse primer to delete ZorAB from pGM196 by KLD.
pGM340	GATTATTCAGGAGTAAGAG	Forward primer to delete ZorEfrom pGM196 by KLD.
	TCTAGAGTCGACCCCTCG	Reverse primer to delete ZorE from pGM196 by KLD.
pGM341	TCTAGAGTCGACCCCTCG	Forward primer to clone the coding sequence of ZorA, followed by ZorB fused to a TEV site and 2xStrepII tag and ZorE fused to a His6x tag in pGM336, under the control of Zorya II native promoter NEBuilder HiFi

		DNA Assembly. Primer linearises vector.
	TTTGATACCTGTGTAAATAATGG	Reverse primer to clone the coding sequence of ZorA, followed by ZorB fused to a TEV site and 2xStrepII tag and ZorE fused to a His6x tag in pGM336, under the control of Zorya II native promoter NEBuilder HiFi DNA Assembly. Primer linearises vector.
	CATTATTTACACAGGTATCAAA ATGTTAGCGCAGCTTTTTGAG	Forward primer to clone the coding sequence of ZorA, followed by ZorB fused to a TEV site and 2xStrepII tag and ZorE fused to a His6x tag in pGM336, under the control of Zorya II native promoter NEBuilder HiFi DNA Assembly. Primer linearises vector. Insert amplified from pGM41.
	GGGTCGACTCTAGAGGATCC GTGATGGTGATGGTGATGCAATTTTG	Reverse primer to clone the coding sequence of ZorA, followed by ZorB fused to a TEV site and 2xStrepII tag and ZorE fused to a His6x tag in pGM336, under the control of Zorya II native promoter NEBuilder HiFi DNA Assembly. Primer linearises vector. Insert amplified from pGM41
pGM342	TCTAGAGTCGACCCCTCG	Forward primer to clone the coding sequence ZorB fused to a TEV site and 2xStrepII tag followed by ZorE fused to a His6x tag in pGM336 by NEBuilder HiFi DNA Assembly. Primer binds to vector to linearise it

	TTTGATACCTGTGTAAATAATGG	Reverse primer to clone the coding sequence ZorB fused to a TEV site and 2xStrepII tag followed by ZorE fused to a His6x tag in pGM336 by NEBuilder HiFi DNA Assembly. Primer binds to vector to linearise it
	CATTATTTACACAGGTATCAAA ATGGATAAGATTATAGGGAAAC	Forward primer to clone the coding sequence ZorB fused to a TEV site and 2xStrepII tag followed by ZorE fused to a His6x tag in pGM336 by NEBuilder HiFi DNA Assembly. Insert amplified from pGM289
	GGGTCGACTCTAGAGGATCC GTGATGGTGATGGTGATGCAATTTTG	Reverse primer to clone the coding sequence ZorB fused to a TEV site and 2xStrepII tag followed by ZorE fused to a His6x tag in pGM336 by NEBuilder HiFi DNA Assembly. Insert amplified from pGM289
pGM343	TCTAGAGTCGACCCCTCG	Forward primer to clone the coding sequence of ZorA followed by ZorE fused to a His6x tag in pGM336 by NEBuilder HiFi DNA Assembly. Primer binds to vector to linearise it
	TTTGATACCTGTGTAAATAATGG	Reverse primer to clone the coding sequence of ZorA followed by ZorE fused to a His6x tag in pGM336 by NEBuilder HiFi DNA Assembly. Primer binds to vector to linearise it
	CATTATTTACACAGGTATCAAA ATGTTAGCGCAGCTTTTTGAG	Forward primer to clone the coding sequence of ZorA followed by ZorE fused to a His6x tag in pGM336 by NEBuilder HiFi DNA Assembly. Insert amplified from pGM290

	GGGTCGACTCTAGAGGATCC GTGATGGTGATGGTGATGCAATTTTG	Reverse primer to clone the coding sequence of ZorA followed by ZorE fused to a His6x tag in pGM336 by NEBuilder HiFi DNA Assembly. Insert amplified from pGM290
pGM344	TCTAGAGTCGACCCCTCG	Forward primer to clone the coding sequence of ZorE fused to a His6x tag in pGM336 by NEBuilder HiFi DNA Assembly. Primer binds to vector to linearise it
	TTTGATACCTGTGTAAATAATGG	Reverse primer to clone the coding sequence of ZorE fused to a His6x tag in pGM336 by NEBuilder HiFi DNA Assembly. Primer binds to vector to linearise it
	CATTATTTACACAGGTATCAAA ATGAAATTATCTATCGAC	Forward primer to clone the coding sequence of ZorE fused to a His6x tag in pGM336 by NEBuilder HiFi DNA Assembly. Insert amplified from pGM291
	GGGTCGACTCTAGAGGATCC GTGATGGTGATGGTGATGCAATTTTG	Reverse primer to clone the coding sequence of ZorE fused to a His6x tag in pGM336 by NEBuilder HiFi DNA Assembly. Insert amplified from pGM291.
