# Supplementary material for: Modularity of Zorya defense systems during phage inhibition
Source: Nat Commun. 2025 Mar 8;16:2344. doi: 10.1038/s41467-025-57397-2 (PMC11890865; doi:10.1038/s41467-025-57397-2)
Supplement: Supplementary file 2 — Description of Additional Supplementary Files [file 41467_2025_57397_MOESM2_ESM.pdf]

Description of Additional Supplementary Files:

Supplementary Data 1: Cblaster output reporting the chromosomal location of Zorya I subtypes

Supplementary Data 2: Cblaster output reporting the chromosomal location of Zorya II subtypes

Supplementary Data 3: Cblaster output reporting the chromosomal location of Zorya III subtypes

Supplementary Data 4: Results of statistical analysis for Zorya I and Zorya II point mutants in Figure 2 and Supplementary Figure 2. Statistical significance for each panel was calculated with Graphpad applying a one-way ANOVA with Dunnett's multiple comparison test
