# Supplementary material for: Modularity of Zorya defense systems during phage inhibition
Source: Nat Commun. 2025 Mar 8;16:2344. doi: 10.1038/s41467-025-57397-2 (PMC11890865; doi:10.1038/s41467-025-57397-2)
Supplement: Supplementary file 4 — Reporting Summary [file 41467_2025_57397_MOESM4_ESM.pdf]

nature portfolio

Corresponding author(s): Giuseppina Mariano
Justin C. Deme
Susan M. Lea

Last updated by author(s): 2025-1-29

Reporting Summary

Nature Portfolio wishes to improve the reproducibility of the work that we publish. This form provides structure for consistency and transparency in reporting. For further information on Nature Portfolio policies, see our Editorial Policies and the Editorial Policy Checklist.

Statistics

For all statistical analyses, confirm that the following items are present in the figure legend, table legend, main text, or Methods section.

n/a	Confirmed	
☐	☒	The exact sample size (*n*) for each experimental group/condition, given as a discrete number and unit of measurement
☐	☒	A statement on whether measurements were taken from distinct samples or whether the same sample was measured repeatedly
☐	☒	The statistical test(s) used AND whether they are one- or two-sided<br>*Only common tests should be described solely by name; describe more complex techniques in the Methods section.*
☒	☐	A description of all covariates tested
☒	☐	A description of any assumptions or corrections, such as tests of normality and adjustment for multiple comparisons
☐	☒	A full description of the statistical parameters including central tendency (e.g. means) or other basic estimates (e.g. regression coefficient) AND variation (e.g. standard deviation) or associated estimates of uncertainty (e.g. confidence intervals)
☐	☒	For null hypothesis testing, the test statistic (e.g. *F*, *t*, *r*) with confidence intervals, effect sizes, degrees of freedom and *P* value noted<br>*Give P values as exact values whenever suitable.*
☒	☐	For Bayesian analysis, information on the choice of priors and Markov chain Monte Carlo settings
☒	☐	For hierarchical and complex designs, identification of the appropriate level for tests and full reporting of outcomes
☒	☐	Estimates of effect sizes (e.g. Cohen's *d*, Pearson's *r*), indicating how they were calculated

Our web collection on statistics for biologists contains articles on many of the points above.

Software and code

Policy information about availability of computer code

Data collection	Data were collected in counted mode in EER format on a CFEG-equipped Titan Krios G4 (Thermo Fisher Scientific) operating at 300 kV with a SelectrisX imaging filter with slid width 10 eV and a Falcon 4 camera
Data analysis	Fiji Java 6, Relion 3.1,cryosparc 3.3, PHENIX 1.20, ChimeraX v1.6.111,CheckMyMetal, Coot 0.9.7, AL2CO , cblaster v 1.3.18 (https://github.com/gamcil/cblaster). MUSCLE v3.8.1551, TrimAL(https://vicfero.github.io/trimal/), IQTree-2 v2.3.6 ,iTOL<br><br>Programs were used as indicated in methods<br>Figures were prepares using Prism GraphPad9 and Adobe Illustrator 2024 and 2025

For manuscripts utilizing custom algorithms or software that are central to the research but not yet described in published literature, software must be made available to editors and reviewers. We strongly encourage code deposition in a community repository (e.g. GitHub). See the Nature Portfolio guidelines for submitting code & software for further information.

Data

Policy information about availability of data

All manuscripts must include a data availability statement. This statement should provide the following information, where applicable:

- Accession codes, unique identifiers, or web links for publicly available datasets
- A description of any restrictions on data availability
- For clinical datasets or third party data, please ensure that the statement adheres to our policy

Cryo-EM volumes and atomic models have been deposited to the EMDB and PDB (accession codes EMD-43560, EMD-43561, EMD-43562, EMD-43563, 8VVN, 8VVI). ZorA and ZorB alignments and structure validation reports are available at https://github.com/GM110Z/Zorya-paper. DNA gels used for quantification of ZorE nickase activity are provided at 10.6084/m9.figshare.28319225. All the remaining data generated in this study and necessary for interpretation are provided in the Supplementary Information and/or Source Data file. Source data are provided with this paper.

Research involving human participants, their data, or biological material

Policy information about studies with human participants or human data. See also policy information about sex, gender (identity/presentation), and sexual orientation and race, ethnicity and racism.

Reporting on sex and gender	N/A
Reporting on race, ethnicity, or other socially relevant groupings	N/A
Population characteristics	N/A
Recruitment	N/A
Ethics oversight	N/A

Note that full information on the approval of the study protocol must also be provided in the manuscript.

Field-specific reporting

Please select the one below that is the best fit for your research. If you are not sure, read the appropriate sections before making your selection.

☒ Life sciences ☐ Behavioural & social sciences ☐ Ecological, evolutionary & environmental sciences

For a reference copy of the document with all sections, see nature.com/documents/nr-reporting-summary-flat.pdf

Life sciences study design

All studies must disclose on these points even when the disclosure is negative.

Sample size	No Sample size calculation was performed
Data exclusions	No exclusions were performed for in vivo work. For cryoEM, particles were excluded from final analysis using pre established classification methods within the softwares and packages described in Methods. Briefly, 2D classification was used to exclude classes that represented contamination, whilst 3D classification was used to remove particles that did not belong to clearly defined structural elements. Details of this process are provided in Methods, Supplementary Figure 8-9-10
Replication	All in vivo experiments were done in 3 biological replicates. All replicates were successful.<br>Protein purification was performed at least three times for each sample and was reproducible.<br>CryoEM datasets were collected for each protein preparation and produced interpretable volumes.<br>All nuclease assays involving purified ZorE were performed 3 times . All gels used for quantification are provided
Randomization	No randomization was performed. N/A for protein and cryoEM
Blinding	N/A as no humans or animals were used

Reporting for specific materials, systems and methods

We require information from authors about some types of materials, experimental systems and methods used in many studies. Here, indicate whether each material, system or method listed is relevant to your study. If you are not sure if a list item applies to your research, read the appropriate section before selecting a response.

Materials & experimental systems

n/a	Involved in the study
☐	☒ Antibodies
☒	☐ Eukaryotic cell lines
☒	☐ Palaeontology and archaeology
☒	☐ Animals and other organisms
☒	☐ Clinical data
☒	☐ Dual use research of concern
☒	☐ Plants

Methods

n/a	Involved in the study
☒	☐ ChIP-seq
☐	☒ Flow cytometry
☒	☐ MRI-based neuroimaging

Antibodies

Antibodies used	Anti-His antibody (1:6000, Cat # MA1-21315, Invitrogen), Anti-Strep (1:10,000, Cat #34850, QIAGEN), Anti-CsrA(1:2000, cat# CSB-PA543019HA01ENT-100ug, Cliniscience), Anti-OmpC(1:2000, Cat # OACA04538-50UG, Cliniscience), Anti-Rabbit (1:10,000, Cat #1706515, Biorad), Anti-Mouse, (1:10,000, Cat #1706516, Biorad)
Validation	Antibodies used were all commercially available and previously validated

Plants

Seed stocks	N/A
Novel plant genotypes	N/A
Authentication	N/A

Flow Cytometry

Plots

Confirm that:

☒ The axis labels state the marker and fluorochrome used (e.g. CD4-FITC).

☒ The axis scales are clearly visible. Include numbers along axes only for bottom left plot of group (a 'group' is an analysis of identical markers).

☒ All plots are contour plots with outliers or pseudocolor plots.

☒ A numerical value for number of cells or percentage (with statistics) is provided.

Methodology

Sample preparation	Sample preparation is described in methods
Instrument	FACS LRS Fortessa (BD)
Software	FlowJo v10.4.2 (Treestar Inc.)
Cell population abundance	For all experiments, 50.000 events were collected
Gating strategy	Gating strategy is shown in Supplementary Figure 7

☒ Tick this box to confirm that a figure exemplifying the gating strategy is provided in the Supplementary Information.
